# Asymmetric Synthesis of 2-Arylethylamines: A Metal-Free Review of the New Millennium

**DOI:** 10.3390/molecules29235729

**Published:** 2024-12-04

**Authors:** Alejandro Manchado, Ángel García-González, Carlos T. Nieto, David Díez, Narciso M. Garrido

**Affiliations:** Department of Organic Chemistry, Faculty of Chemical Sciences, University of Salamanca, Pl. Caídos, s/n, 37008 Salamanca, Spain; alex92mc@usal.es (A.M.); u149349@usal.es (Á.G.-G.); eneas@usal.es (C.T.N.); ddm@usal.es (D.D.)

**Keywords:** 2-phenethylamine, 2-arylethylamine, asymmetric synthesis, chiral induction, organocatalysis, organophotocatalysis, enzymatic catalysis

## Abstract

2-Arylethylamines are presented in several natural bioactive compounds, as well as in nitrogen-containing drugs. Their ability to surpass the blood–brain barrier makes this family of compounds of especial interest in medicinal chemistry. Asymmetric methodologies towards the synthesis of 2-arylethylamine motives are of great interest due to the challenges they may present. Thus, a concise metal-free review presenting recent advances in the asymmetric synthesis of 2-arylethylamines is presented, covering last-millennium studies, considering different methodologies towards the aforementioned motif, including chiral induction, organocatalysis, organophotocatalysis and enzymatic catalysis.

## 1. Introduction

**Figure 1:**
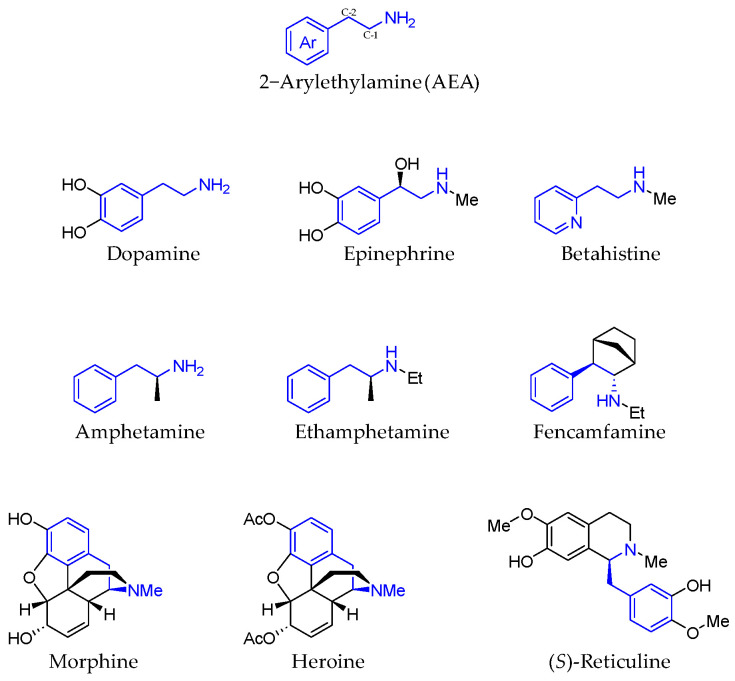
2-Arylethylamine derivatives.

2-Phenethylamine motif is present in several bioactive compounds, both natural and human-made ones. From dopamine or adrenaline to morphine, 2-phenethylamine skeletons are present in multiple drugs because of their well-known properties (Figure 1) [1,2]. Thanks to their relatively small size, they can go through the blood–brain barrier, being the main endogenous catecholamines affecting dopaminergic neurons, playing an important role in voluntary movement stress or mood [3].

We have already reported two interesting studies reviewing the importance of this family of compounds in medicinal chemistry for both 2-phenethylamines [4] and 2-heteroarylamines [5], covering the medicinal chemistry landscape considering drugs incorporating the aforementioned moiety including different substitutions, functional groups, and ring enclosures.

Also, previous reviews have been reported in this field, covering different topics when it comes to arylethylamine (AEA) synthesis. In 2022, Xiaowei et al. [6] published a review about the asymmetric photocatalytic synthesis of azaarene derivatives, and in 2023, Moran et al. [7] reported a review considering both metal and metal-free synthetic methodologies towards β-(hetero)arylethylamines. Chiral amine synthesis was properly covered by both Zhou et al. in 2010, [8] and Riera et al. in 2022 [9], considering transition metal-catalysis towards enantioselective hydrogenation and by Rueping et al. in 2023 [10] covering enzymatic catalysis. Also, the functionalization of alkenes has been proved to be a successful field towards the synthesis of AEA, and a couple of reviews were reported about this field, in 2020 by Studer et al. [11] and in 2022 by Wang et al. [12] and by Miura et al. [13].

In this sense, the lack of metal-free asymmetric synthesis of AEA in the literature is remarkable, and we believe that the present review will be of great help for the scientific community.

Although the biosynthesis of this family is a well-known subject as they have been deeply studied [2,3,14,15,16,17], laboratory synthesis is still a rather complex topic, although it has been showing interesting advances coming from quite different approaches, resulting in an interesting variety of reactions capable of creating the aforementioned motif. Among them, the asymmetric synthesis of 2-arylethylamines (AEA) seems to generate the most interest due to the complicity of developing chiral moieties similarly to biosynthetic pathways, as the useful physiological activity of pharmaceuticals containing asymmetric centers almost always appears in only one enantiomer.

**Figure 2:**
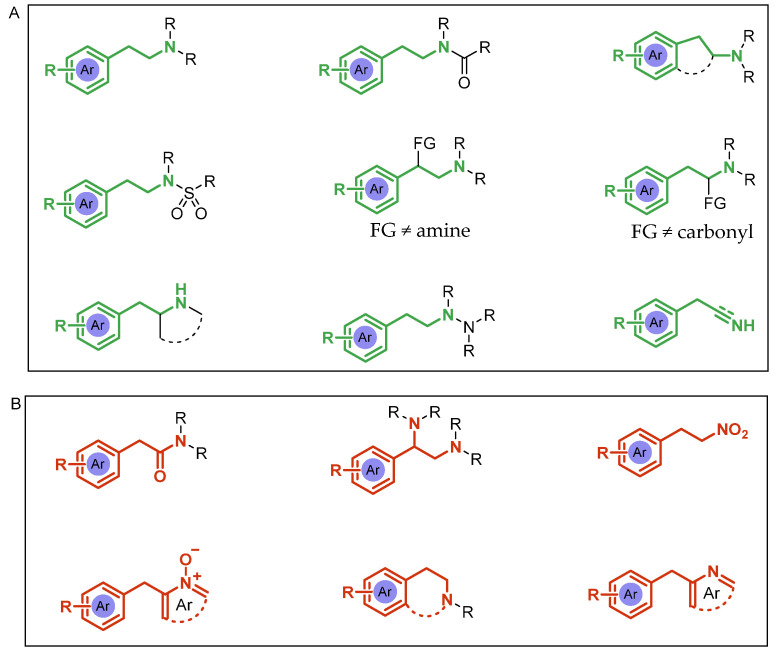
(**A**)**:** 2-Arylethylamines covered in this review. (**B**): 2-Arylethylamines not covered in this review.

Thus, we present a metal-free review by reporting the main advances in the last millennium in the chiral synthesis of 2-arylethylamines. This review covers the asymmetric synthesis of AEA skeletons considering different substituents, functional groups, and aryl/heteroaryl rings and their combinations, such as those shown in Figure 2A. Chirality is considered either in C-1, C-2, or in both carbon atoms. Also, different 2-phenethylamine-containing moieties are not covered, as shown in Figure 2B.

This review is organized based on the type of chemistry used to produce the corresponding chiral AEA skeleton, considering the chiral introduction process as the key step in the synthesis, although subsequent transformations can be carried out in order to synthesize the corresponding amine. Either yield, ee, dr, or all of them are discussed as the main objective method to compare methodologies. Also, subsequently, applications of the studied products are discussed. Thus, this review is structured as follows: chiral induction, organocatalysis, organophotocatalysis, and enzymatic catalysis.

## 2. Chiral Induction

Although the chiral induction-based asymmetric synthesis of AEA has not seen the same number of advances in recent time than other methods that are discussed in the following, it still is a very important tool when it comes to the development of new chiral drugs, either through a prochiral substrate which can be transformed into a chiral compound due to the introduction of a chiral reactive, or through a chiral substrate that can evolve to a desired chiral product; thus, most recent advances in this field are presented.

**Scheme 1:**
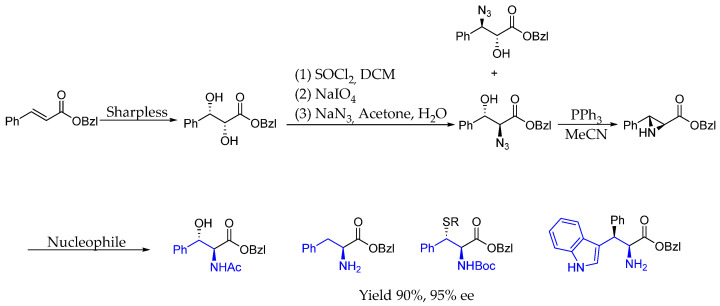
Chiral α-amino acid containing AEA synthesized by Sharpless chiral induction and subsequent derivatization.

In 2002, Hruby et al. [18] developed a methodology to obtain β-substituted tryptophan, cysteine, and serine derivatives from prochiral α,β-unsaturated carbonyls. Chirality was achieved with Sharpless asymmetric dihydroxylation as the key step, producing enantiopure aziridines, which, in subsequent reactions, could undergo stereospecific and regioselective ring-opening processes, leading to the aforementioned chiral AEA with both excellent yield and ee (Scheme 1).

**Scheme 2:**
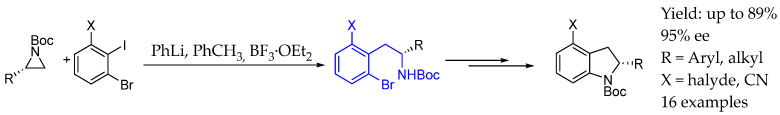
Chiral AEA and derived dihydroindoles obtained by Dineen et al.’s methodology [19].

Later on, in 2009, Dineen et al. [19] achieved the ring opening of chiral aziridines with a great degree of success when it came to yields and ee. Aryl iodide substrates containing *o*-bromo substituents were found to properly react with asymmetric aziridines in the presence of organolithium compounds and boron trifluoride, finding phenyllithium as a proper reagent providing a more general method, synthesizing a great variety of enantiopure AEA in great yields and ee. Further derivatizations through Buchwald’s palladium-catalyzed intramolecular amination reaction gave access to 2-substituted dihydroindoles (Scheme 2).

**Scheme 3:**
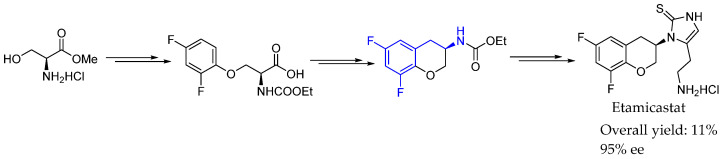
Multikilogram-scale synthesis of etamicastat.

In 2012, Beliaev et al. [20] achieved an interesting methodology to synthesize etamicastat, a dopamine β-hydroxylase inhibitor, on a multikilogram scale. A convergent manufacturing route was developed and scaled up to 60 kg. A key step was the synthesis of chiral 3-aminochroman intermediate and 2-aminoethyl imidazolethione, which were combined in order to synthesize the corresponding bioactive compound. From optically active L-SerOMe HCl, affordable [chiral pool] and easy to synthesize by the asymmetric synthesis of α-amino acid methodologies, convenient *O*-alkylation and *N*-alkylation, followed by intramolecular Friedel–Craft ring closing and subsequent hydrogenation, led to the desired chiral AEA in great yields and ee (Scheme 3).

**Scheme 4:**
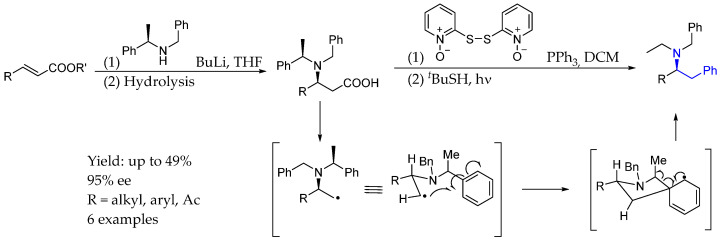
Different chiral AEAs and plausible 1,4-phenyl radical rearrangement mechanism from Garrido et al. [21].

Then, in 2018, Garrido et al. [21] presented an unprecedent methodology to develop chiral arylethylamines, involving a prochiral substrate and an asymmetric aza-Michael addition, a key step to chiral introduction. A novel 1,4-phenyl radical rearrangement domino process starting in a Barton decarboxylation reaction of the corresponding chiral β-amino acids led to the aforementioned products, with moderate yield and excellent ee. This rearrangement involved cyclisation by the dearomatization of the aryl group and subsequent rearrangement by rearomatization, leading to chiral AEA (Scheme 4).

**Scheme 5:**
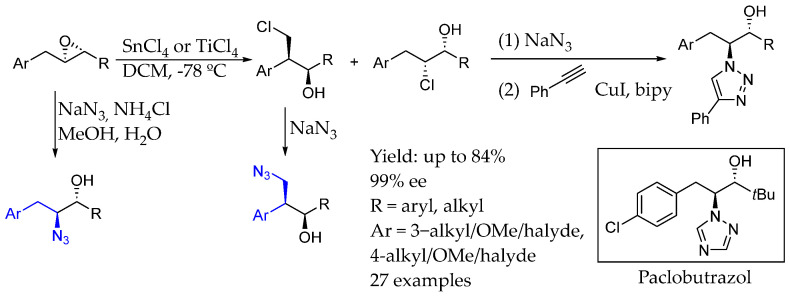
Chiral AEA and paclobutrazol derivative accessible from Race et al.’s methodology [22].

Following this field, in 2020, Race et al. [22] developed a synthesis starting from chiral epoxide as a substrate in order to obtain AEA and used it to synthesize a paclobutrazol analog, a plant growth regulator, with an excellent yield and ee. This methodology involved the selective and regiodivergent opening of unsymmetrical phenonium ions. Although different regioselective chlorination could be achieved by changing the Lewis acid, chiral AEA could also be synthesized by the sodium azide ring opening of epoxide substrates (Scheme 5).

**Scheme 6:**
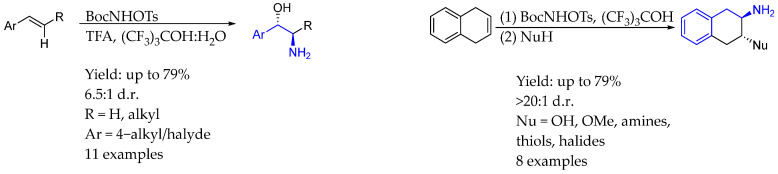
Different diastereoselective AEA syntheses from Bower et al. [23].

In 2024, Bower et al. [23] synthesized a big variety of AEA through a regioselective process involving stereospecific 1,2-aminohydroxylation under acidic conditions, promoted by BocNHOTs, starting from alkene substrates, either styrenyl or aliphatic alkenes, leading to a great dr. This methodology could provide unprotected 1,2-aminoalcohols by intermolecular aza-Prilezhaev aziridination followed by stereospecific SN_2_ opening by water. Also, by replacing water with other nucleophile, 1,2-amino(thio)etherification, diamination, aminoazidation, and aminofluorination reactions could be carried out in order to obtain interesting products (Scheme 6).

## 3. Organocatalysis

Chiral auxiliaries are frequently used in order to introduce chirality to prochiral substrates. Ecofriendly processes are in demand nowadays, as offering methods involving environmentally friendly and efficient catalysts, able to deliver high yields with limited byproducts within short reaction times under mild conditions, showing a broad substrate scope to allow the construction of diverse molecular structures, involving non-toxic catalysts, potent and generic modes of activation, mild conditions, and high concentrations that reduce waste and solvent volumes should be the main objective of current chemistry [24]. These challenging demands can be solved by the use of organocatalysts, a name given by MacMillan in 2000 [25], which have experienced great development in recent years, even resulting in a Nobel prize given to Benjamin List and David MacMillan in 2021 [26] for the development of asymmetric organocatalysis. Organocatalysts must bind to either the substrate or the reactive to produce an asymmetric intermediate that can evolve to the desired chiral product. Thus, organocatalysts can be shortened depending on which element or functional group the interaction is based on. When it comes to chiral AEA synthesis, lately, the development has been based on these types of organocatalysts: thioureas, phosphoric acid and phosphonates, amines, amides and ammonium species, aryl iodine species, aldehydes, boron species, and selenium species.

### 3.1. Thioureas

The replacement of the oxygen atom of urea by sulfur results in a higher acidity and increased strength in the hydrogen bond donation [27]. Thioureas are also known to possess a unique non-covalent dual hydrogen-bond donor ability, playing an important role in catalysis. Bifunctional thioureas have proved to be excellent organocatalysts when it comes to enantioselective C-C bond-forming reactions [28,29].

**Scheme 7:**
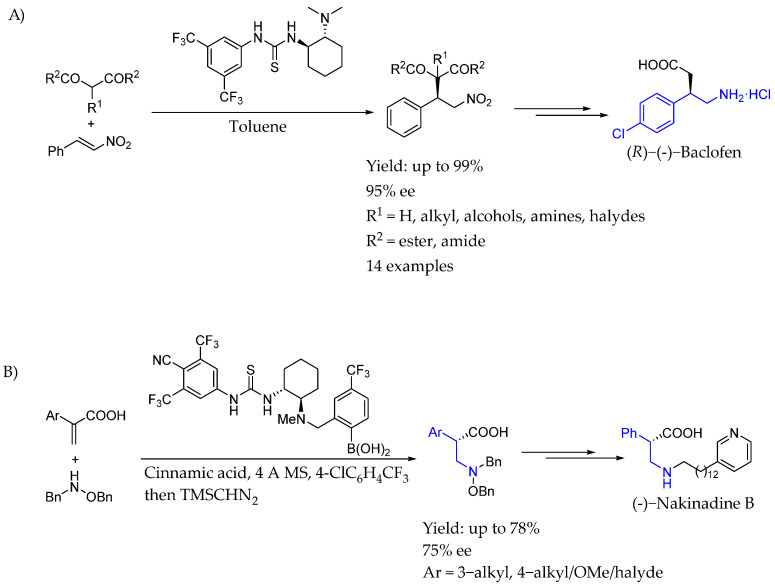
(**A**) Use of bifunctional chiral urea organocatalysis for the synthesis of different nitro-compounds and their application towards the synthesis of chiral AEA (*R*)-(-)-Baclofen by Takemoto et al. in 2005 [30]. (**B**) Synthesis of chiral α-carboxyl AEA by Takemoto et al. in 2021 and its application towards the synthesis of (-)-Nakinadine B.

In 2005, Takemoto et al. [30] developed an asymmetric Michael addition of 1,3-dicarbonyl compounds to nitroolefins catalyzed by a bifunctional thiourea. They prepared and tested a variety of novel bifunctional organocatalysts and demonstrated that thiourea moiety and amino group of the catalyst activated a nitroolefin and a 1,3-dicarbonyl compound, respectively, leading to the synthesis of chiral AEA (*R*)-(-)-Baclofen, an inhibitory neurotransmitter in the central nervous system of mammalians, with an excellent yield and ee (Scheme 7A). Later, in 2021 [31], they also used a thiourea–boronic acid hybrid catalyst to introduce chirality in an aza-Michael addition to α,β-unsaturated carboxylic acids, leading to an interesting variety of chiral AEAs with an excellent yield and ee. This efficient method, which could control carbonyl α-chirality with functionalizing β-position by the conjugate addition-asymmetric protonation thanks to bifunctional chiral thiourea–boronic acid organocatalyst, was able to produce interesting biologically active compounds by a subsequent transformation of the directly synthesized substrates (Scheme 7B).

**Scheme 8:**
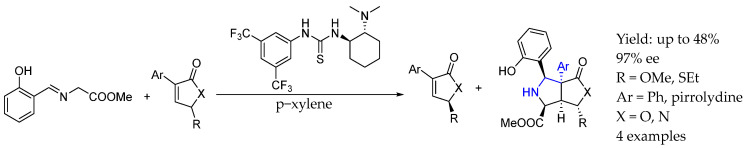
DKR to obtain polysubstituted chiral AEA enabled by bifunctional urea organocatalysis by Alemán et al. [32].

In 2024, Alemán et al. [32] achieved a classic kinetic resolution of furanone derivatives by an asymmetric cycloaddition enabled by a chiral bifunctional thiourea–amine organocatalyst, deeply studying the process through successful DFT studies. The resulting product was a highly substituted AEA in which the amine group was embedded into a cyclopentane-fused ring. This methodology involved a highly enantioselective organocatalyzed [3 + 2] cycloaddition of furanone derivatives and azomethine ylides, leading to highly multifunctional bicyclic adducts with five stereogenic centers. In combination with the kinetic resolution of butenolides, achieving selectivity factors of above 200, furan-2(5*H*)-ones and furo [3,4-*c*]pyrrolidinones were obtained in high yields and ee (Scheme 8).

### 3.2. Phosphorous-Based Species

By Brönsted acid-type organocatalysis, phosphorous-based organocatalyst relies on proton transfer to the substrate, via either double hydrogen-bonding interactions or synergetic hydrogen-bonding and ion-pairing interactions [32,33].

**Scheme 9:**
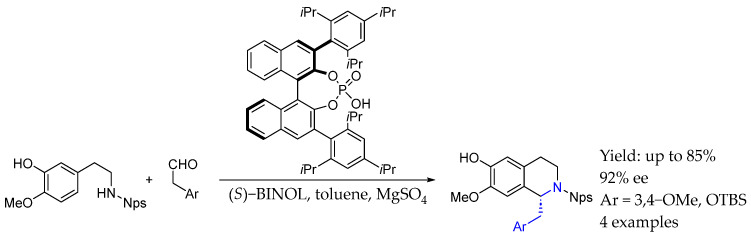
Chiral tetrahydroisoquinoline containing AEA synthesized by phosphorous organocatalysis by Hiemstra et al. [34].

In 2015, Hiemstra et al. [34] used a chiral binapthtyl phosphate organocatalyst to develop an asymmetric Pictet–Spengler reaction towards the synthesis of *N*-(*O*-nitrophenylsulfenyl)-2-arylethylamines with arylacetaldehydes, resulting in 1-benzyl-1,2,3,4-tetrahydroisoquinolines substrates. In this process, AEA, in which the amine was embedded into a tetrahydroisoquinoline scaffold, great yields and ee were achieved. The resulting optically active products could be derivatized by varying the aryl group in order to obtain interesting bioactive compounds (Scheme 9).

**Scheme 10:**
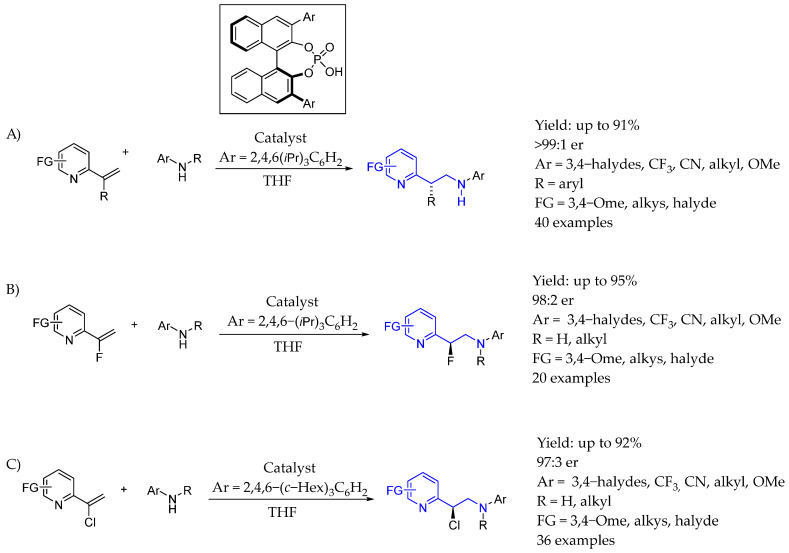
Chiral AEA synthesis, enabled by phosphorous organocatalyst, either with α-aryl substituents (**A**), α-F substituents (**B**), or α-Cl substituents (**C**) by Watson et al. in 2018, 2020, and 2022, [35,36,37] respectively.

Some years later, in 2018, Watson et al. [35] synthesized a vast number of chiral azaheteroaryl ethylamines using an asymmetric binaphthyl phosphoric acid that promoted a dearomatizing aza-Michael addition towards prochiral exocyclic aryl enamine, which underwent asymmetric protonation and rearomatization, leading to a great ee (Scheme 10A). Two years later, in 2020 [36], they developed a methodology to introduce chirality into a C-F bond, starting from the corresponding fluorinated substrates and using the aforementioned asymmetric binaphthyl phosphoric organocatalyst. By the same dearomatizing aza-Michael addition and asymmetric protonation upon rearomatization mechanism, vicinal fluoramines could be obtained with great yields and ee (Scheme 10B). Following this research, in 2022 [37], they also achieved the same level of success by using the corresponding chlorinated substrates, synthesizing interesting chiral heterocyclic vicinal chloramines by varying substituents in the corresponding organocatalyst moiety. This methodology relied upon the asymmetric protonation of a catalytically generated aryl chloroenamine using a chiral Brønsted acid, resulting in asymmetric AEA with excellent yields and ee (Scheme 10C).

**Scheme 11:**
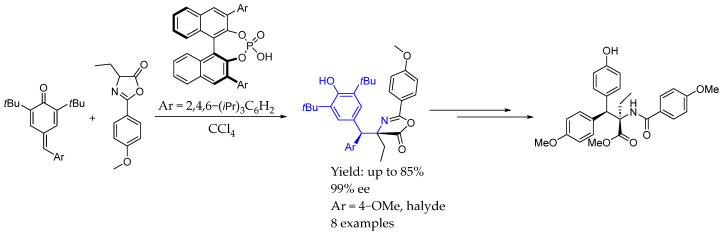
Li et al.’s methodology towards the synthesis of β,β-diaryl-α-amino acid esters [38].

Back to 2018, Li et al. [38], using the same phosphoric acid-based catalyst, also achieved really good numbers regarding the yield and ee, leading to more complex molecules by the 1,6-addition of azlactones to *p*-quinone methides. The mechanism of this methodology relied on the activated transition state in which the bifunctional organocatalyst bound to both carbonyl groups, enabling the 1,6-addition. The corresponding cyclic substrates could be derivatized in order to obtain interesting β,β-diaryl-α-amino acid esters bearing adjacent tertiary and quaternary stereogenic centers (Scheme 11).

**Scheme 12:**
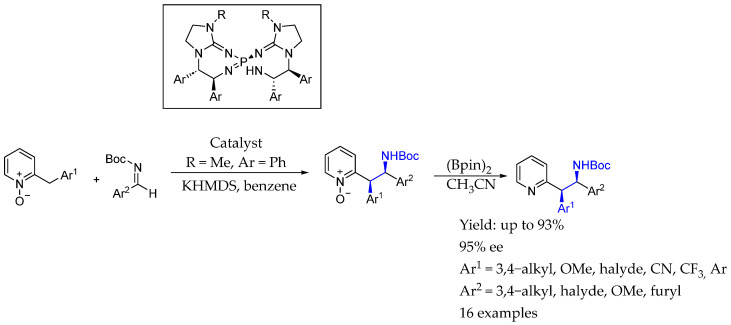
Chiral synthesis of pyridine containing AEA by Terada et al. [39].

Also in 2018, Terada et al. [39] developed a synthesis of 2-benzylpyridine *N*-oxides, which could be transformed into AEA in which the nitrogen atom was embedded into a pyridine scaffold, by an iminophosphorane superbase, resulting in a highly functionalized compound with a great ee. The process used a chiral bis(guanidino)iminophosphorane to overcome the issue of the less acidic pronucleophile *N*-oxides to carry out the diastereo- and enantioselective direct Mannich-type reaction with *N*-Boc imines (Scheme 12).

**Scheme 13:**
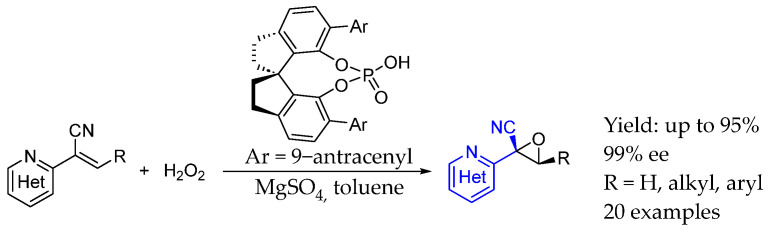
Chiral oxirane containing AEA enabled by phosphorous organocatalyst, from Mao et al. in 2024 [40].

Finally, in 2024, Mao et al. [40] developed an asymmetric epoxidation of alkenyl aza-heteroarenes using a chiral phosphoric acid as organocatalyst. In this case, the AEA moiety was already present as substrates and chirality was introduced by the epoxidation. This technique achieved high enantio- and diastereoselectivity and also great chemo- and stereocontrol during the epoxidation of alkenyl aza-heteroarenes. By both electrostatic and hydrogen-bonding interactions, substrates as well as hydrogen peroxide were activated, resulting in very interesting chiral oxetanes able to be diversified in order to obtain *N*-heterocyclic frameworks used in both pharmaceutical development and synthetic chemistry (Scheme 13).

### 3.3. Amines, Amides, and Ammonium Species

Nitrogen-based organocatalysis relies on the basicity and nucleophilicity of nitrogen species. Carbonyl or phosphate activation and direct deprotonation are the main pathways in order to perform a chiral transfer from the organocatalyst into the prochiral substrate.

**Scheme 14:**
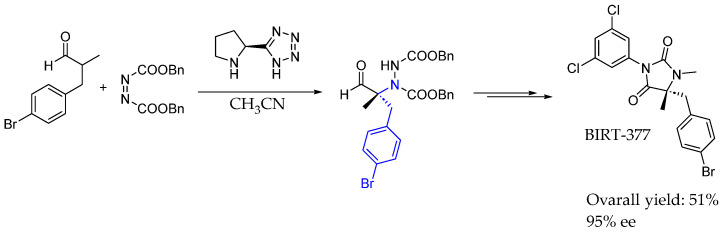
Total synthesis of BIRT-377 using L-proline-derived catalyst by Barbas et al. in 2005 [41].

In 2005, Barbas et al. [41] used L-proline-derived tetrazole-catalyzed asymmetric synthesis to introduce chirality in a methodology capable of producing the total synthesis of an LFA-1 antagonist, BIRT-377, synthesizing highly functionalized AEA in the process. Proline insertion and hydrogen bond interaction were responsible for the direct asymmetric α-amination of 3-(4-bromophenyl)-2-methylpropanal with dibenzyl azodicarboxylate, synthesizing quaternary amino acid in the process (Scheme 14).

**Scheme 15:**
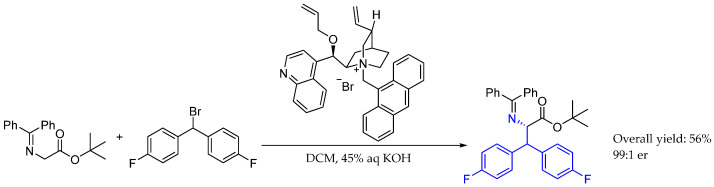
Asymmetric synthesis of L-phenylalanine derivative by Patterson et al. in 2007 [42].

In 2007, Patterson et al. [42] developed a chiral synthesis of phenylalanine derivative using the asymmetric phase-transfer catalyzed alkylation of *tert*-butyl glycinate–benzophenone Schiff base by a cinchona alkaloid-derived catalyst. In order to scale the process up to multiple kilograms, it was necessary to add the catalyst or the base last. Both great yield and ee could be achieved by the α-asymmetric deprotonation enabled by the asymmetric ammonium-based organocatalyst (Scheme 15).

**Scheme 16:**
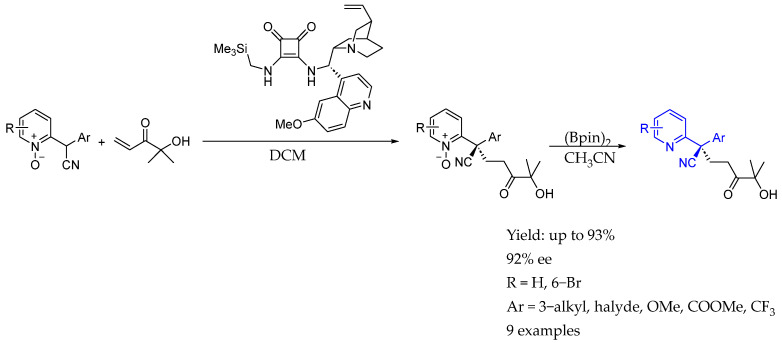
Synthesis of chiral AEA whose nitrogen is embedded into a CN group, from Izquierdo et al. in 2016 [43].

Almost ten years later, in 2016, Izquierdo et al. [43] used a bifunctional chiral squaramide-based organocatalyst towards the Brønsted base-catalyzed conjugate addition of substituted 2-cyanomethylpyridine (and pyrazine) *N*-oxides to acrylate equivalents for the direct asymmetric α-functionalization of 2-alkyl aza-arenes, producing interesting 2-*tert*-alkyl aza-aryl adducts, finding the *N*-oxide functionality of substrates that acted as a removable activating and stereodirecting group These products could be derivatized in order to obtain the corresponding AEA with great yields and ee (Scheme 16).

**Scheme 17:**
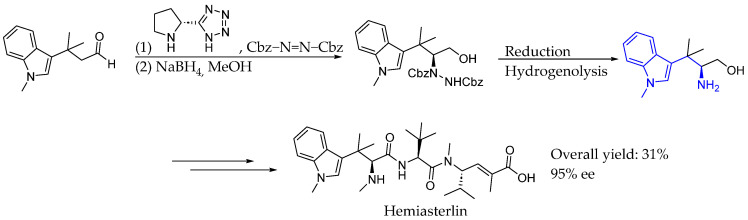
Total synthesis of hemiasterlin by Lindal et al. in 2017 [44].

Also in 2017, Lindel et al. [44] achieved the total synthesis of hemiasterlin, a powerful cytotoxic marine natural product. The tetramethyltryptophan moiety was assembled by the *tert*-prenylation of indole, followed by the high-yielding organocatalyzed α-hydrazination of sterically constricted aldehyde in excellent enantioselectivity. A key step in chiral induction was the use of L-proline-based tetrazole as the asymmetric organocatalyst. It is worth mentioning that the same organocatalyst and nitrogen source were also used by Barbas et al. in 2005 [41], achieving the same degree of success. Thus, producing chiral AEA as a key intermediate to the synthesis of the desired natural product, the subsequent development of the adducts could lead to hemiasterlin by peptide coupling (Scheme 17).

**Scheme 18:**
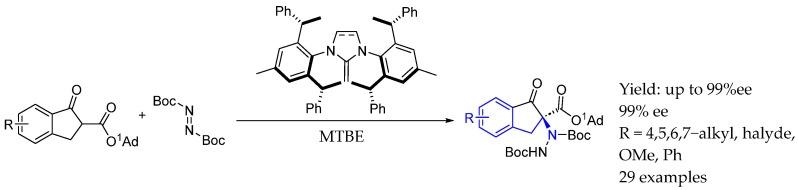
*N*-heterocyclic olefin bifunctional organocatalyst used by Dong et al. in 2022 [45] for the synthesis of chiral AEA.

Later on, in 2022, Dong et al. [45] used chiral *C*2-symmetric *N*-heterocyclic olefin (NHOs) bifunctional organocatalyst in order to obtain interesting highly functionalized AEA by the asymmetric α-amination of β-ketoesters. Electron-rich and highly polarized C=C double bond, enabled by the donating property of nitrogen atoms, resulted in the strong basicity and high nucleophilicity of the exocyclic carbon. Thus, this bifunctional organocatalyst mechanism relied on base and dual hydrogen-bonding bifunctional activation, providing excellent stereoselectivities (Scheme 18).

**Scheme 19:**
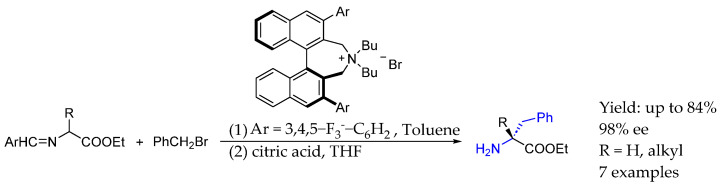
Asymmetric synthesis of AEA by Liu et al. in 2024 [46].

More recently, in 2024, Liu et al. [46] developed an asymmetric synthesis of chiral AEA based on the asymmetric phase-transfer alkylation of *N*-(arylmethylene)-α-alkylamino acid ethyl esters and *N*-(arylmethylene)glycine ethyl esters. The introduction of chirality was achieved by using a simplified Maruoka catalyst, based on asymmetric binaphtyl ammonium derivative. Products obtained through this methodology could be derivatized in order to obtain interesting bioactive compounds, such as Olodanringan, an angiotensin II type 2 receptor antagonist (Scheme 19).

**Scheme 20:**
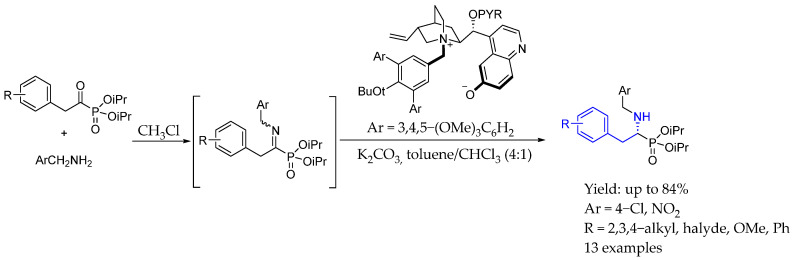
Chiral aminophosphonate-based AEA by Deng et al. in 2024 [47].

Simultaneously, Deng et al. [47] achieved the complex synthesis of chiral AEA based on α-aminophosphonates by a ppm-loading cinchone-derivative organocatalyst, able to produce a total turnover number of 20,000−1,000,000, achieving enzyme-like efficiency via a network of weak bonding interactions that effectively guided the substrate and catalyst toward a transition-state-like complex. This highly efficient asymmetric isomerization of α-iminophosphonates led to an interesting variety of enantiopure compounds with excellent yields and ee (Scheme 20).

**Scheme 21:**
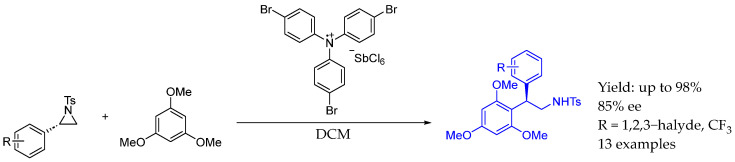
Magic-blue-enabled synthesis of chiral AEA by Ghorai et al. in 2024 [48].

Finally, Ghorai et al., also in 2024 [48], reported the synthesis of chiral AEA starting from the corresponding optically active activated aziridine by developing a methodology consisting of a highly stereospecific synthetic approach to Friedel−Crafts-type alkylation of arenes/heteroarenes enabled by a magic blue-initiated S_N_2-type ring-opening reaction. In this stereospecific process, an interesting asymmetric AEA could be created with good yields and ee (Scheme 21).

### 3.4. Aryl Iodine Species

The main advances in the synthesis of chiral AEA by using asymmetric aryl iodide organocatalysts come from their ability to activate double bonds by iodine insertion, generating chiral intermediates able to evolve into desired products.

**Scheme 22:**
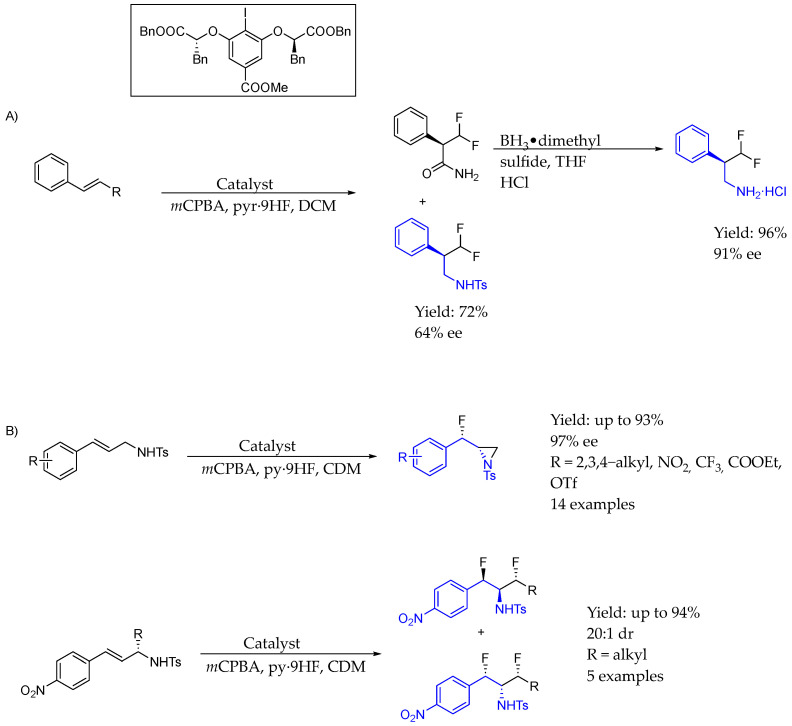
(**A**) Chiral aryl iodide organocatalyst used in the synthesis of 1,1-di-fluorinated AEA. (**B**) Synthesis of 1,3-di-fluorinated AEA.

In 2016, Jacobsen et al. [49] used a chiral aryl iodide catalyst in order to synthesize enantiopure AEA, reporting a method for the catalytic, asymmetric, migratory geminal difluorination of β-substituted styrenes by direct reaction with styrene substrates or the late transformation of the corresponding chiral aryl ethyl amide, resulting with excellent yields and ee. The reaction used a chiral aryl iodide catalyst, showing that cation–π interactions played important role in stereodifferentiation and could be carried out on a multigram scale (Scheme 22A). Also, two years later, in 2018 [50], they used the same methodology with styrene derivatives that included an NHT group to produce interesting chiral AEA in which the amine group was embedded into an aziridine scaffold, by catalytic diastereo- and enantioselective fluoroamination enabled by the chiral aryl iodide activation of the corresponding double bond, producing either 1-fluoro-2-aziridine derivatives or 1,3-difluoro-2-amine derivatives bearing two or three contiguous stereocenters, respectively (Scheme 22B).

### 3.5. Aldehydes and Ketones

Aldehyde and ketone organocatalysis rely on the ability to activate amino-containing molecules, producing a reactive chiral intermediate by condensation able to evolve to asymmetric AEA. Either from oxaziridine or imine/iminium intermediates, the carbonyl affinity of amino compounds is used towards the synthesis of chiral AEA.

**Scheme 23:**
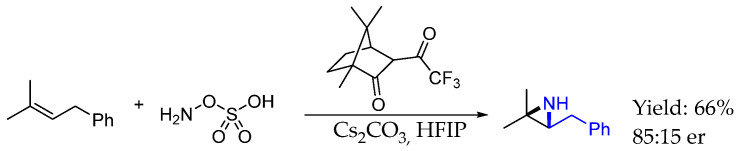
Chiral AEA obtained through Kürt et al.’s methodology in 2020 [51].

In 2020, Kürti et al. [51] reported the synthesis of interesting aziridine-containing chiral AEA under mild conditions by using an organocatalytic nitrogen transfer methodology. This methodology could provide the aziridination of isolated C=C bonds with good yields and ee by a nitrogen transfer from hydroxylamine-*O*-sulfonic acid as a nitrogen source and electron-deficient ketones as catalysts, affording direct access to unprotected aziridines with selectivity that could not be achieved otherwise (Scheme 23).

**Scheme 24:**
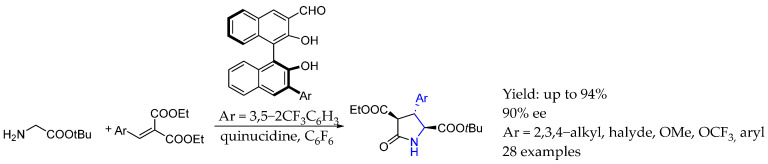
Chiral pyrrolidinone-containing AEA synthesized by Wen et al. in 2022 [52].

Interestingly, in 2022, Wen et al. [52] developed a synthesis of enantiopure AEA based on pyroglutamic acid derivatives. That novel methodology was promoted by a chiral aldehyde-induced tandem conjugated addition–lactamization, using an asymmetric binaphthyl-based aldehyde. That methodology relied on the amino insertion into the catalyst carbonyl group to create an activated intermediate in which the α-deprotonation and subsequent addition took place, resulting in great yields and ee (Scheme 24).

**Scheme 25:**
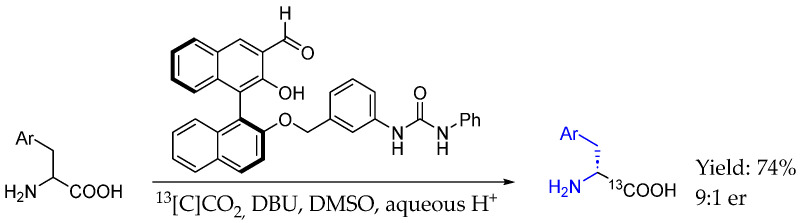
^13^C labelling of chiral AEA by Lundgren et al. in 2024 [53].

Finally, in 2024, Lundgren et al. [53] described the first example of an enantioselective carbon isotope exchange reaction resulting in (radio)labeled α-amino acids, enabled by a bifunctional chiral aldehyde receptor with isotopically labeled CO_2_ followed by an imine hydrolysis. That methodology relied on the condensation between the α-amino acid and chiral aldehyde, generating the corresponding imine which underwent deprotonation to form a reactive enolate intermediate which was hydrogen-bonding to itself through the urea linker, resulting in [^13^C]COOH amino acid-based chiral AEA with excellent yields and ee (Scheme 25).

### 3.6. Boron Species

Acting as Lewis acids, the organoboron-based asymmetric synthesis of AEA’s main methodology relies on boronyl radicals to activate proper substrates.

**Scheme 26:**
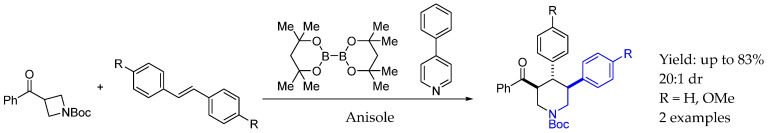
Piperidine-containing chiral AEA enabled by boron organocatalyst from Zhang et al. in 2024 [54].

Recently, in 2024, Zhang et al. [54] developed a regio- and diastereoselective synthesis of polysubstituted piperidines by the radical [4 + 2] cycloaddition of 3-aroyl azetidines and various alkenes, including previously unreactive 1,2-di-, tri-, and tetrasubstituted alkenes, enabled by commercially available diboron compounds and 4-phenylpyridine. In this highly selective process, the key step was the radical ring opening of the azetidine ring. Thus, asymmetric AEA in which the nitrogen atom was embedded in a piperidine ring produced a high yield and dr (Scheme 26).

### 3.7. Selenium Species

Usually, organoselenium catalysts are based on the production of selenenylated synthetic intermediates, followed by oxidation and elimination, as their electrophilic characteristics make organoselenium compounds great alkene activators [55,56].

**Scheme 27:**
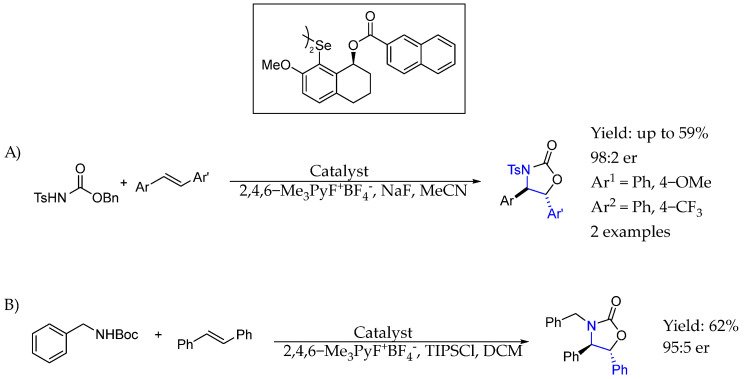
Selenium organocatalyst used by Denmark et al. [57] to produce chiral AEA. (**A**) *N*,*N*’-bistosyl urea are used, as the bifunctional nucleophile. (**B**) *N*-Boc amines are used.

In 2019, Denmark et al. [57] used a chiral selenium-based organocatalyst together with an *N*,*N*’-bistosyl urea as the bifunctional nucleophile and *N*-fluorocollidinium tetrafluoroborate as the stoichiometric oxidant to obtain the *syn*-diamination of alkenes enantioselectively. Interestingly, that methodology was also able to afford *syn*-oxyamination, leading to highly functionalized chiral AEA with high yields and ee (Scheme 27A). Later on, in 2024 [58], they used the same methodology to synthesize 2-oxazolidinones from *N*-Boc amines and mono or *trans-*disubstituted alkenes, generating a variety of chiral AEA with high enantioselectivities. They found that critical to the success of the transformation was the inclusion of triisopropylsilyl chloride, likely because it sequestered fluoride generated by the boronate oxidant throughout the reaction and suppressed side reactivity (Scheme 27B).

## 4. Organophotocatalysis

The use of visible light to promote organic transformations has shown interesting and rapid advances in the last decades. When it comes to waste management and reaction efficiency, light seems to offer great advantages for use at both laboratory and industry scales [59]. In this sense, progress in this field has allowed researchers to produce ideal, cheap, and energy-efficient light sources, available for photocatalysis. Although ultraviolet light has been used in classical photochemistry, visible light, as well as photoredox catalysis and photosensitizers, is much easier to use in a typical laboratory setup [60].

Since the report by MacMillan et al. in 2008 on visible-light singly occupied molecular orbital (SOMO) photoredox catalysis allowing enantioselective α-alkylations by a combination of photoredox catalysis and organocatalysis [61], photoredox catalysis using visible light has been applied to a large range of important organic transformations [62,63,64,65].

Organophotocatalysts on organic dyes can be excited by light and this way activate intermediates either by electro, hydrogen, or energy transfer. The most recent use of different photocatalyst with or without combination with other techniques in organic synthesis are discussed in the following.

**Scheme 28:**
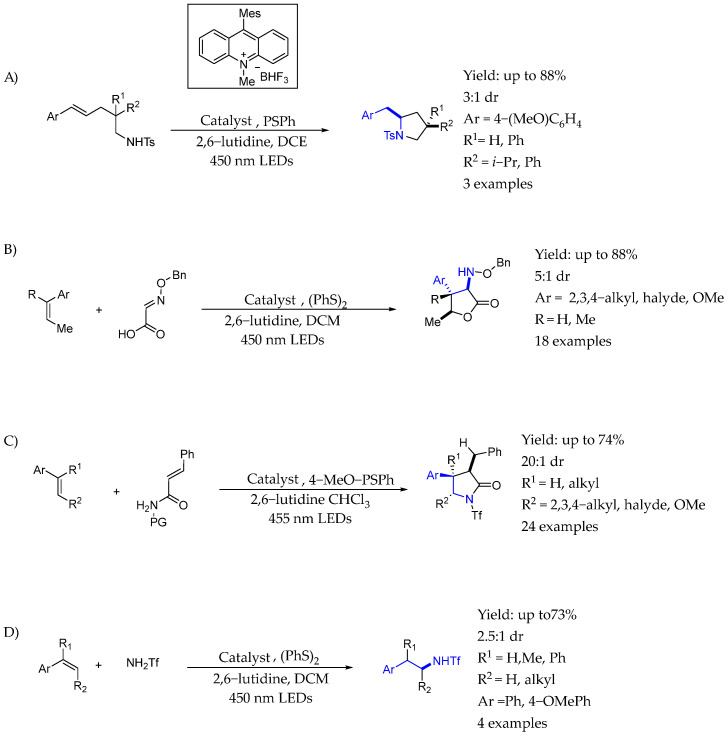
Different chiral AEA arrays synthesized by Nicewicz et al. [66] (**A**) Intramolecular anti-Markonikov hydroamination. (**B**) Radical cycloaddition to produce γ-lactams and (**C**) pyrrolidines. (**D**) Intermolecular anti-Markonikov hydroamination.

Nicewicz et al. reported an array of anti-Markovnikov alkene hydrofunctionalization reactions employing a dual catalyst system consisting of an acridinium photoredox catalyst in conjunction with a redox-active hydrogen atom donor or surrogate (aryl disulfide). This general catalyst system enabled several important reactions in this area, including anti-Markovnikov alkene hydroacetoxylation, hydrolactonization, hydroamination, and hydrotrifluoromethylation reactions. In 2013 [66], they reported an intramolecular anti-Markovnikov hydroamination method using 9-mesityl-10-methylacridinium tetrafluoroborate and thiophenol as a hydrogen-atom donor with visible light (Scheme 28A). The reaction conditions were mild and affected a range of cyclization modes to give important nitrogen-containing heterocycles. Interesting chiral AEAs in which the nitrogen atom was embedded into a pyrrolidine ring were synthesized in great yields and moderate dr. The mechanism involved a single electron transfer enabled by an excited photocatalyst, resulting in an intramolecular ring closing reaction. The hydrogen atom transfer from thiophenol produced the desired products. Two years later, in 2015 [67,68], they also demonstrated that that protocol could be extended to intermolecular reactions (Scheme 28B,C). In this way, they presented a mild, efficient synthetic method to access γ-lactams and pyrrolidines heterocycles from alkenes and activated unsaturated amides or protected unsaturated amines, respectively, through a photoredox-mediated approach by polar radical crossover cycloadditions from simple oxidizable alkenes and unsaturated amides nucleophiles. Using a mesityl acridinium single electron photooxidant and a thiophenol cocatalyst under irradiation, they were able to directly synthesize these important classes of heterocycles with complete regiocontrol. A year later [69], they hoped to be able to accomplish an *anti*-Markovnikov-selective intermolecular alkene hydroamination with simple amines and alkenes without the need for prefunctionalized starting materials, and after examining a variety of ammonia surrogates, they found that TfNH_2_ afforded the highest yield of *anti*-Markovnikov hydroamination with β-methylstyrene using 20 mol % thiophenol as the cocatalyst, furnishing the expected addition product with an isolated yield of 87%. Trisubstituted alkenes were able to create products with modest diastereoselectivity (Scheme 28D).

**Scheme 29:**
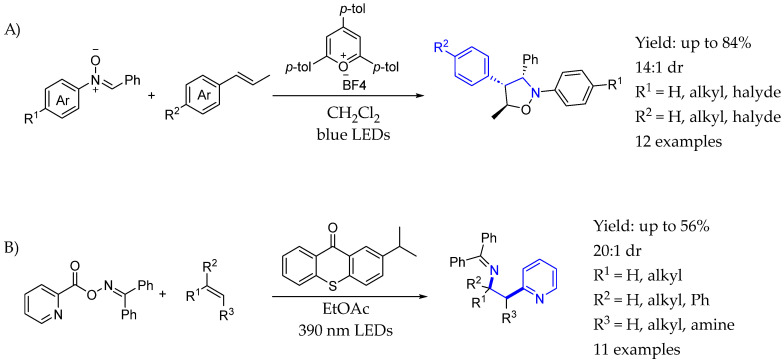
Asymmetric synthesis of AEA through organophotocatalysis by Xia et al. [70] (**A**) Nitrone 1,3-dipolar addition. (**B**) Intermolecular amino(hetero)arylation.

In 2017, Xia et al. [70] reported photoredox reactions of 1,3-dipolar cycloaddition of nitrones with alkenes. That methodology, in which the nitrones were cyclized with oxidizable styrenes and aliphatic alkenes via a polar radical crossover cycloaddition reaction through a photocatalytic reaction without additives, was *anti*-regioselective to the typical 1,3-dipolar nitrone cycloaddition reactions. Thus, it offered an efficient synthetic method to obtain isoxazolidine derivatives under mild conditions, thus obtaining an interesting chiral AEA in the process. An excited photocatalyst was responsible for the single electron transfer with the corresponding styrenyl substrates, which underwent a nitrone addition and a subsequent intramolecular ring-closing reaction, creating the desired products after a chain propagation mechanism involving styrenyl substrates as single electron transfer agents (Scheme 29A). Later on, in 2023 [71], they also reported a metal-free and photoinduced intermolecular amino(hetero)arylation reaction for the single-step installation of both (hetero)aryl and iminyl groups across alkenes in an efficient and regioselective manner. An interesting chiral AEA could be synthesized by a photoinduced energy transfer methodology, enabled by a thianoxantone photocatalyst. The mechanism relied on the energy transfer from excited photocatalyst to the corresponding iminosubstrates, followed by decarboxylation and pyridine fragmentation, which underwent an addition onto the corresponding alkene substrates followed by an imine insertion, creating the desired products with a good yield and excellent dr (Scheme 29B).

**Scheme 30:**
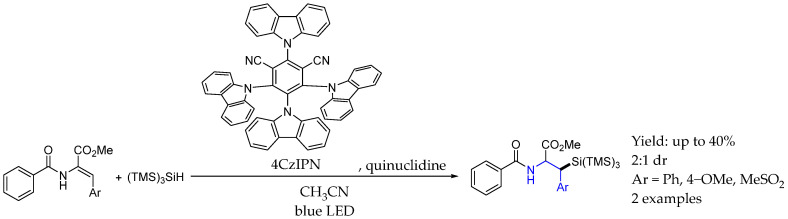
Chiral (TMS)_3_Si containing 2-arylethylacetamides by Jing et al. in 2023 [72].

Later, in 2023, Jing et al. [72] developed an efficient metal-free method for synthesizing unnatural β-silyl-α-amino acids via hydrosilylation. That protocol allowed for the direct hydrosilylation of dehydroalanine derivatives, leading to moderate to good yields of β-silyl-α-amino acids. The visible-light-induced reaction employed commercially available hydrosilanes and was characterized by its mild conditions and high atom economy. The mechanism relied on the hydrogen atom transfer from (TMS)_3_SiH towards activated quinuclidine, enabling substrate activation by radical formation, followed by a single electron transfer from an excited photocatalyst, which had previously undergone a single electron transfer in order to activate quinuclidine. This way, polysubstituted chiral AEAs were synthesized with moderate yields and dr. Also, they applied that methodology in order to synthesize interesting key intermediates in the synthesis of Sphingofungin E, an antifungal agent (Scheme 30).

**Scheme 31:**
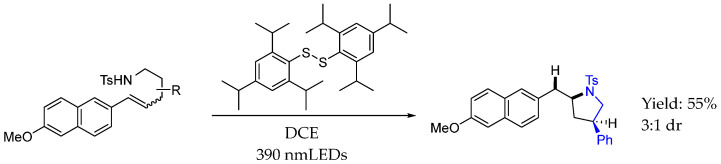
Hydroamination of olefins to produce asymmetric AEA by Zeng et al. in 2023 [73].

Also in 2023, Zeng et al. [73] developed a novel strategy to achieve the photoinduced *anti*-Markovnikov hydroamination of olefins. The process involved *N*-centered radical generation through N−S bond “forming and cleaving” with bis(2,4,6-triisopropylphenyl) disulfide, an inexpensive reagent with relatively low oxidative ability, as a photo and hydrogen atom transfer catalyst upon irradiation with visible light, to achieve intramolecular hydroamination. The mechanism relied on an energy transfer step from an excited photocatalyst to form an activated intermediate after homolytic S-S bond cleavage and N-S bond formation with the substrates. Then, the homolytic N-S bond cleavage and subsequent *anti*-Markovnikov intramolecular ring-closing reaction and hydrogen atom transfer generated the desired products with a good yield and moderate dr (Scheme 31).

**Scheme 32:**
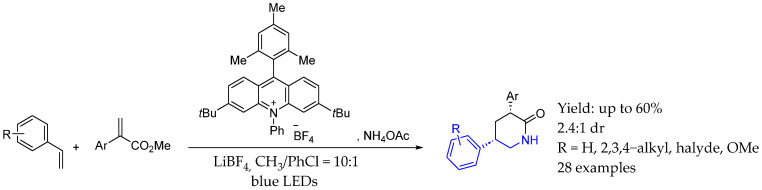
Organophotocatalysis to produce chiral δ-lactam-containing AEA by Shu et al. in 2023 [74].

At the same time, Shu et al. [74] described an organophotocatalyzed [1 + 2 + 3] strategy for the one-step synthesis of diverse substituted 2-piperidinones from accessible inorganic ammonium salts, alkenes, and unsaturated carbonyl compounds. That mild protocol operated at room temperature and demonstrated exclusive chemoselectivity, accommodating both terminal and internal alkenes with various functional groups. The method efficiently constructed two C–N bonds and one C–C bond in a single step, enabled by organophotocatalysis. The mechanism relied on the single electron oxidation of the alkene enabled by the excited photocatalyst, followed by a nitrogen addition, and the subsequent addition to the α,β-unsaturated carbonyl allowed the internal ring-closing reaction by a single electron transfer from the photocatalyst (Scheme 32).

**Scheme 33:**
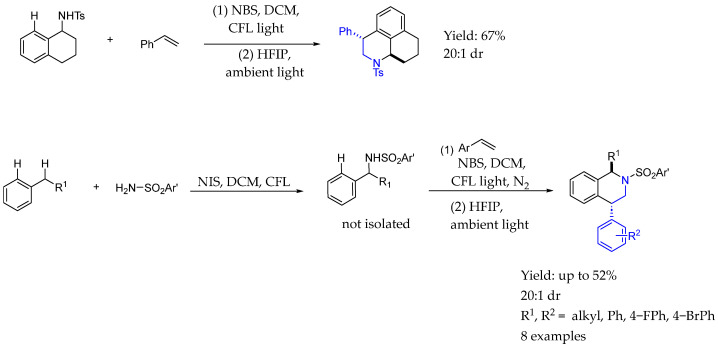
Tetrahydroisoquinoline-containing chiral AEA by Li et al. in 2004 [75].

In 2024, Li et al. [75] reported a versatile strategy for synthesizing a diverse variety of *N*-heterocycles enabled by a multicomponent cascade coupling process, started from NBS. With the utilization of common olefins, that simple protocol facilitated their coupling with various bifunctional reagents. The process relied on controlling the radical addition to olefins, combined with a polar cyclization reaction, in order to achieve great yields and dr (Scheme 33).

## 5. Enzymatic Catalysis

Enzymatic catalysis, also known as biocatalysis, has evolved in the last two decades. In the early 2000s, its main use was the synthesis or resolution of optically active compounds; since then, enzymatic catalysis has been used more and more both at laboratory and industry scales and has been a broadly applicable tool in organic synthesis [76].

Biocatalysis relies on the use of enzymes, which possess a tremendous catalytic activity resulting in higher stereoselectivity, higher yield, higher efficiency, and less waste, as either chiral auxiliaries or frameworks to synthesizes desired products which otherwise would be complex to produce with other classic methods [77]. Despite their well-known potential, their stability and cost are still considered big limitations when using that methodology [78].

Considering the type of reaction catalyzed by enzymes, several approaches have been developed in order to synthesize the aforementioned compounds, including transaminases, amine dehydrogenases, imine reductases, reductive aminases, and amino acid decarboxylases and oxidases, among others.

Herein, recent advances in the use of enzymes as the main tool in the synthesis of chiral AEA are presented.

### 5.1. Transaminases

**Scheme 34:**
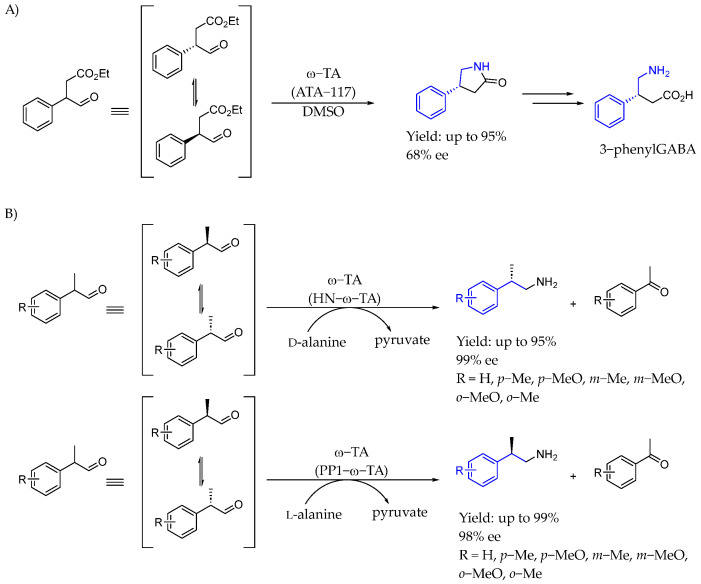
DKR by Kroutil et al. to produce γ-lactam containing chiral AEA in 2009 (**A**) and 1-methyl AEA in 2014 (**B**) [79].

In 2009, Kroutil et al. [79] presented a novel method for synthesizing enantiomerically enriched 4-phenylpyrrolidin-2-one using dynamic kinetic resolution (DKR) through an enzymatic enantioselective amination reaction catalyzed by ω-transaminases, specifically ATA-117. That three-step process, optimized for co-solvent and pH conditions, allowed access to arylpyrrolidin-2-ones derivatives, the cyclic analogs of γ-aminobutyric acid (GABA) derivatives. The approach simplified synthesis by avoiding the cumbersome racemic amine derivatives (Scheme 34A). Then, in 2014 [80], they reported the dynamic kinetic resolution of 2-phenylpropanal derivatives using ω-transaminases. That methodology generated optically active AEA with an enantiomeric excess of up to 99%. Enantiocomplementary ω-transaminases facilitated the production of both (*R*)- and (*S*)-enantiomers, revealing differing stereopreferences from those seen with methyl ketones (Scheme 34B).

**Scheme 35:**
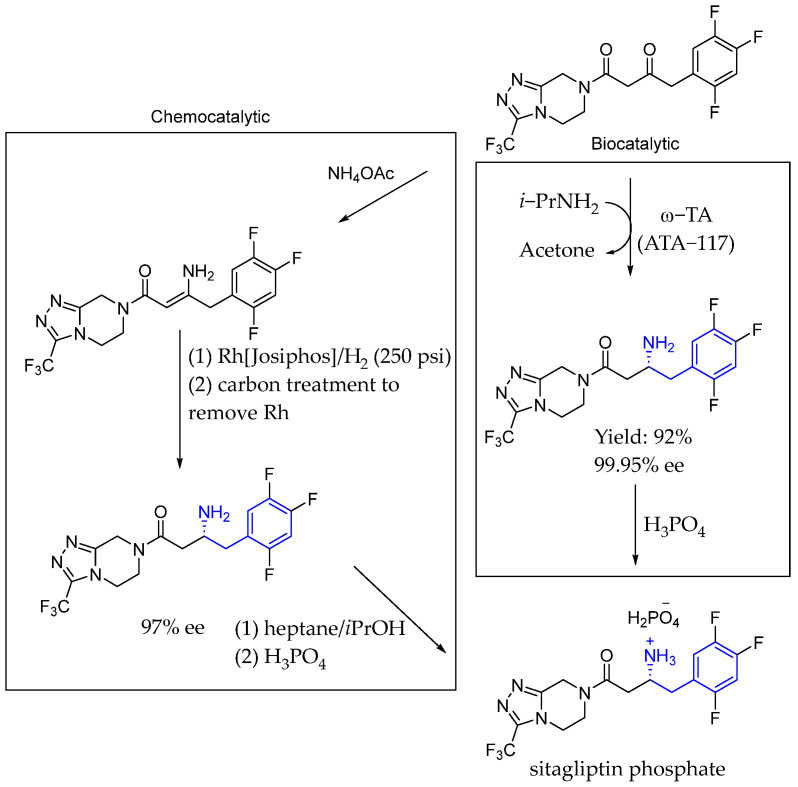
Novel enzymatic process replacement towards the synthesis of sitagliptin phosphate by Savile et al. in 2010 [81].

Back in 2010, Savile et al. [81] reported an efficient biocatalytic process to replace a recently implemented rhodium-catalyzed asymmetric enamine hydrogenation for the large-scale manufacture of the antidiabetic compound sitagliptin, employing a modelling and mutation approach to develop a transaminase with improved activity for synthesizing chiral amines, which were previously only obtainable through resolution methods. Their biocatalytic route featured the direct amination of prositagliptin ketone to provide enantiopure sitagliptin, followed by phosphate salt formation to provide sitagliptin phosphate (Scheme 35).

**Scheme 36:**
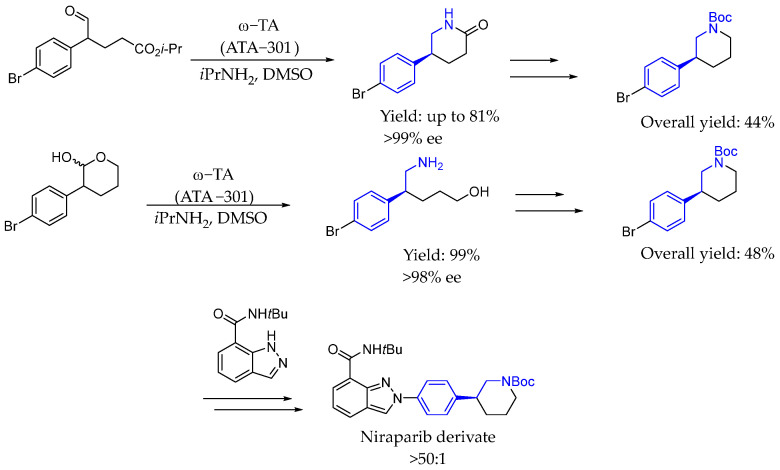
Convergent routes towards the synthesis of niraparib derivative enabled by transaminases by Chung et al. in 2014 [82].

Again in 2014, Chung et al. [82] described new asymmetric routes towards an indazole derivative niraparib. Novel transaminase-mediated dynamic kinetic resolutions of racemic aldehyde surrogates provided enantioselective syntheses of the 3-aryl-piperidine coupling partner. The use of amine transaminase ATA-301 as biocatalyst and isopropyl amine as aminating agent resulted in chiral AEA with an excellent ee and yield (Scheme 36).

**Scheme 37:**
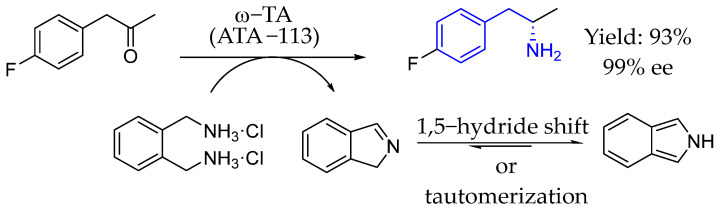
Turner et al.’s synthesis of chiral AEA enabled by transaminase, in 2014 [83].

Also in 2014, Turner et al. [83] developed a highly efficient method for chiral AEA synthesis enabled by ω-transaminase and ortho xylylene diamine as a diamine donor, showing a high conversion without the need for byproduct removal. This simple approach was compatible with both (*R*)- and (*S*)-selective ω-TAs and was particularly effective for substrates with difficult equilibria (Scheme 37).

**Scheme 38:**
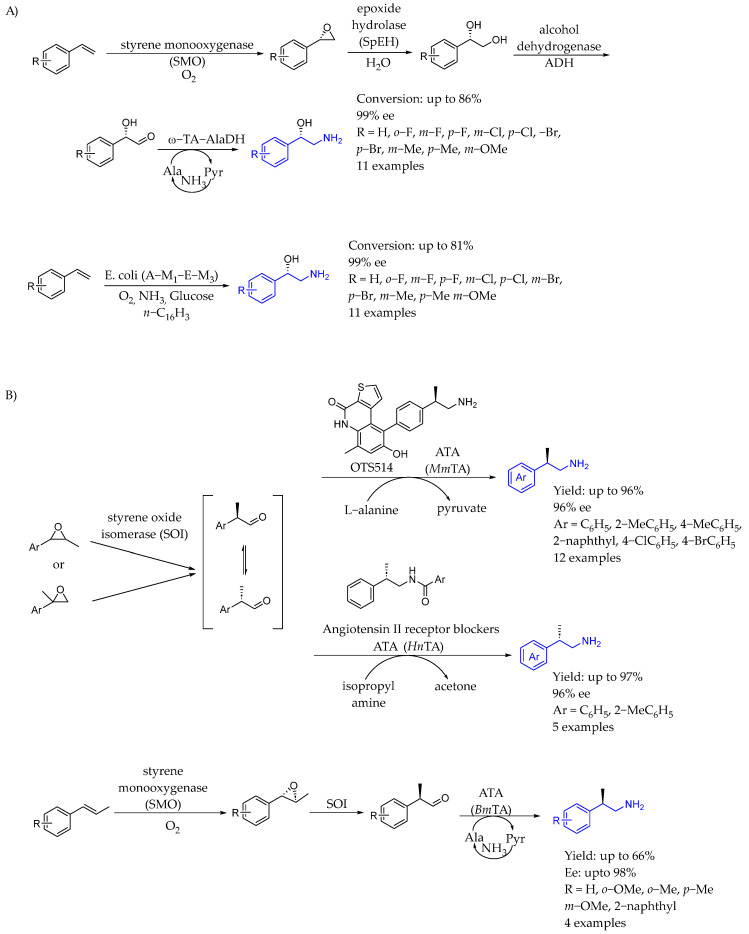
Chiral amino alcohol from styrenyl derivatives (**A**) and epoxide derivatization through chiral AEA (**B**) by Li et al. [84].

In 2016, Ref. [84] Li et al. developed a new type of one-pot asymmetric oxy- and amino-functionalization of terminal alkenes through cascade biocatalysis (Scheme 38A). That modular approach enabled the engineering of efficient one-pot cascade biocatalysis involving more than four concurrent enzymatic reactions, allowing for simple and environmentally friendly syntheses of chiral AEA with high enantiomeric excess and high yields. Although chirality was induced by styrene monooxygenase, ω-transaminase was used in order to obtain the corresponding 1,2-aminoalcohols (Scheme 38A). Years later, in 2022, Refs. [85,86] they developed a new type of one-pot multi-step enzymatic cascade transformation involving dynamic kinetic resolution to convert racemic substrates to chiral products with a high ee and yield (Scheme 38B). The reaction started with in situ aldehyde generation from epoxides racemic trans-β-methyl or α-methyl easily accessible via styrene oxide isomerase (SOI)-catalyzed Meinwald epoxide rearrangement to generate 2-arylpropanal, which was followed by a spontaneous racemization and transaminase-catalyzed (*R*)- or (*S*)-enantioselective amination to produce (*R*)- and (*S*)-2-arylpropyl amines (Scheme 38B).

**Scheme 39:**
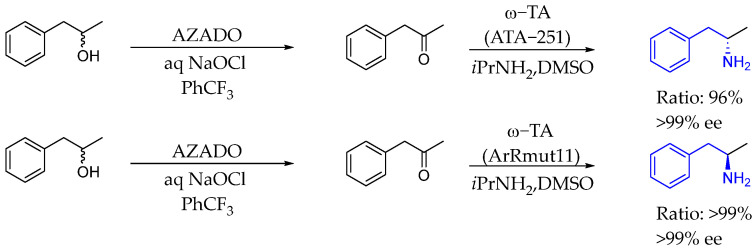
α-Methyl AEA enabled by transaminase catalysis by Rebolledo et al. in 2017 [87].

In 2017, Rebolledo et al. [87] presented a simple and stereoselective protocol for synthesizing optically active AEA, by combining an organocatalyst (AZADO), an oxidant (NaOCl), and an enzyme (ω-transaminase), providing high yields and excellent diastereo- and enantiomeric excess in a one-pot aqueous process starting from racemic alcohols. This methodology represented a robust alternative to existing methods, effectively merging organocatalysis and enzymatic catalysis (Scheme 39).

**Scheme 40:**
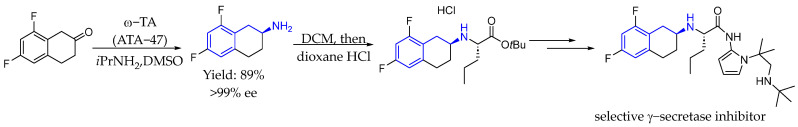
γ-Secretase inhibitor synthesis by Burns et al. in 2017 [88].

Also in 2017, Burns et al. [88] described a chemoenzymatic route for the production of an intermediate to a selective γ-secretase inhibitor with potential antitumor activity, with help from bioinformatics studies. The process employed both a transaminase catalyzed reductive amination of a substituted tetralone and an alcohol dehydrogenase catalyzed reduction of an α-ketoester to generate the two chiral centers in the molecule, with nearly perfect stereoselectivity. Chiral AEA was synthesized with an excellent yield and ee (Scheme 40).

**Scheme 41:**
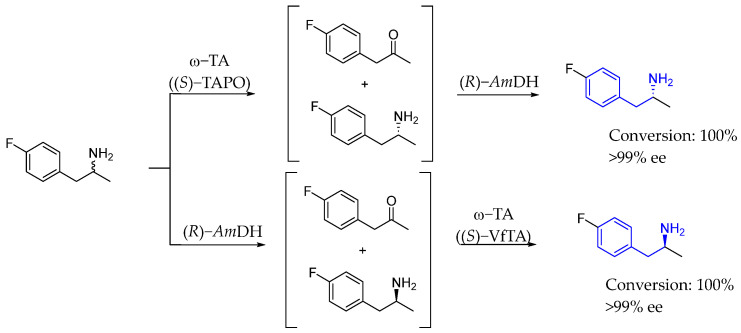
One-pot enantioselective deracemization to produce chiral AEA by Yun et al. in 2019 [89].

Later on, in 2019, Yun et al. [89] introduced a novel one-pot enantioselective deracemization method for α-chiral amines using complementary biocatalysts. That process involved enantioselective deamination enabled either by ω-transaminase or amine dehydrogenase, after which the complementary enzyme acted, generating chiral AEA with excellent yields and ee (Scheme 41).

**Scheme 42:**
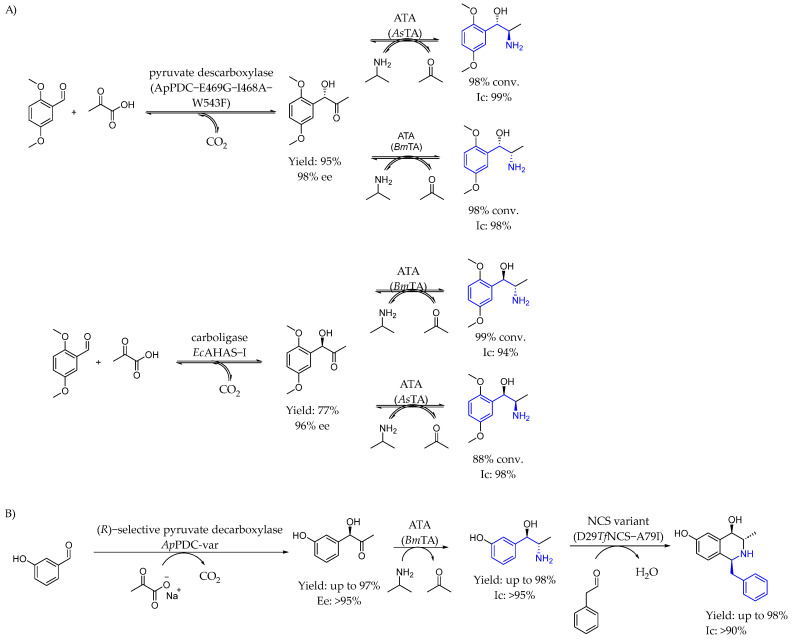
Methodology to produce four amino alcohol enantiomers enabled by carbolygase-transaminase catalysis (**A**) and tetrahydroisoquinoline-containing AEA (**B**) by Rother et al. in 2019 [90] and 2023 [91], respectively.

In 2019, Rother et al. [90] developed a biocatalytic method to produce the four stereoisomers of methoxamine through a sequential one-pot, two-step cascade reaction using pyruvate and 2,5-dimethoxybenzaldehyde without intermediate isolation (Scheme 42A). High conversions and good stereoselectivities were achieved by combining selective carboligases and transaminases. This process included a variant of pyruvate decarboxylase from *Acetobacter pasteurianus*, enabling the production of an intermediate with 98% enantiomeric excess. The *Bacillus megaterium* transaminase was key in aminating sterically demanding ketones, achieving methoxamine stereoisomers with purities up to 99%. A few years later, in 2023 [91], they presented a multi-enzymatic catalyzed process to synthesize a 1,3,4-substituted tetrahydroisoquinoline using purified enzymes and starting materials derived from renewable sources (Scheme 42B). The key step in the synthesis was the production of chiral alcohol-containing AEA enabled by transaminase catalysis. That approach allowed the production of a complex product with three chiral centers in only four highly selective steps. The purified transaminase catalyzed the synthesis of (1*R*,2*S*)-metharaminol with high yields and the subsequent cyclization using norcoclaurin synthase (NCS) led to the formation of the target product, demonstrating a highly efficient stepwise approach to obtain AEA with high yields.

**Scheme 43:**
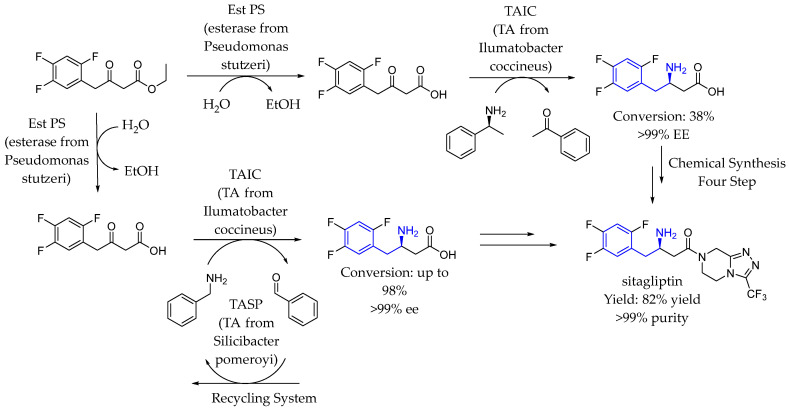
Sitagliptin synthesis by transaminase catalysis by Yun et al. in 2021 [92].

In 2021, Yun et al. [92] reported a bienzymatic one-pot efficient cascade to produce β-amino acids as an intermediate in excellent conversions (82–95%) for the synthesis of the leading oral antidiabetic drug. They developed a whole-cell biotransformation using recombinant *Escherichia coli* coexpressing esterase and transaminase, wherein the desired expression level of each enzyme was achieved by promotor engineering. Finally, sitagliptin phosphate was chemically synthesized from β-amino acids with 82% yield and >99% purity. The same way, a year later, they designed and developed a coupled enzyme cascade to synthesize the sitagliptin intermediate using a two-cell system, effectively avoiding enzyme inhibition and enhancing overall efficiency. The process involved fusing two different transaminases (TAs) to regenerate the amino donor. In the system, a β-keto acid was converted by the first TA into the sitagliptin intermediate using (*S*)-α-MBA as the amino donor. The second TA recycled the deaminated product, acetophenone, as a substrate, regenerating (*S*)-α-MBA. This innovative approach optimized the enzymatic reaction while maintaining high efficiency (Scheme 43).

**Scheme 44:**
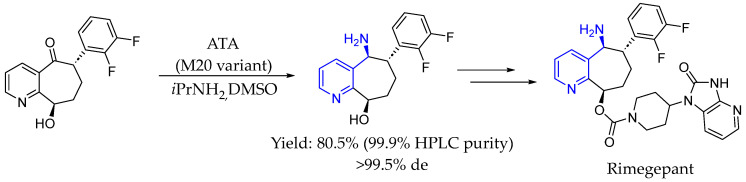
Transaminase catalyzed synthesis of rimegepant intermediate by Jiao et al. [93].

In 2022, Jiao et al. [93] reported the successful engineering of a transaminase for the efficient synthesis of a key intermediate in rimegepant, a CGRP antagonist used to treat migraines. Starting from an enzyme with no initial activity toward the target ketone, the rational design produced a variant with trace activity, ultimately achieving a 99% conversion, a >99.5% enantiomeric excess, and an 80% yield with a 99.9% HPLC purity, demonstrating industrial potential. These improved transaminase variants showed enhanced activity with bulky substrates, enabling efficient biosynthesis in aqueous buffer without metal catalysts, reducing solvent use, and enhancing overall yield (Scheme 44).

**Scheme 45:**
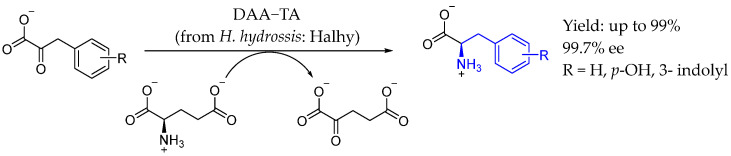
D-amino acid-based AEA by Bezsudnova et al. in 2022 [94].

That year, Bezsudnova et al. [94] studied a D-amino acid transaminase from *Haliscomenobacter hydrossis* as a biocatalyst for producing optically pure D-amino acids from aromatic keto acids. Using D-glutamate as an amino group donor, they created a one-pot, three-enzyme system which included transaminase with two auxiliary enzymes (hydroxyglutarate dehydrogenase and glucose dehydrogenase), which achieved a yield of up to 99% and an enantiomeric excess of over 99% (Scheme 45).

**Scheme 46:**
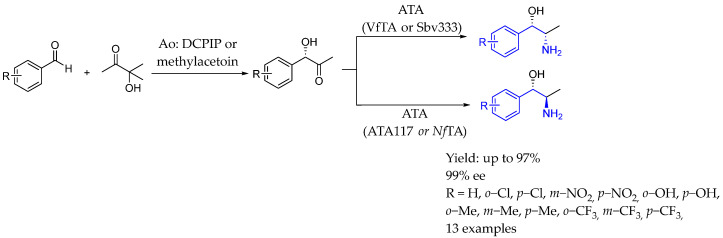
Enzymatic methodology with both (*S*)- and (*R*)-selectivity to produce chiral AEA by Ferrandi et al. in 2024 [95].

In 2024, Ferrandi et al. [95] designed a novel bi-enzymatic synthesis using a two-step carbologation/transamination procedure catalyzed by (*S*)-selective acetoin:dichlor-ophenolindophenol oxidoreductase (DCPIP) and a transaminase with (*S*)- or (*R*)-selectivity for the preparation of Nor(pseudo)ephedrine analogs, achieving acceptable yields and good diastereomeric and enantiomeric excesses (Scheme 46).

**Scheme 47:**
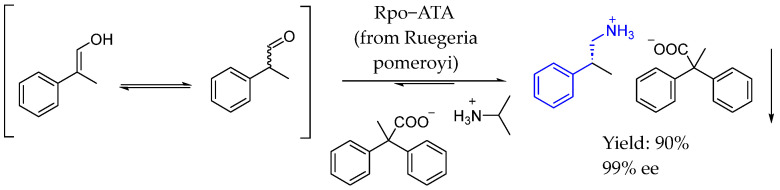
In situ product crystallization of chiral AEA salt by Langermann et al. 2024 [96].

That same year, Langermann et al. [96] investigated the enantioselective synthesis of β-chiral amines through transamination, combining this technique with in situ product crystallization to prevent the formation of unwanted byproducts. Using 2-phenylpropanal as a substrate, enantiomeric excesses of up to 99% of (*R*)-β-methylphenethylamine ((*R*)-2-phenylpropan-1-amine) were achieved with a commercially available transaminase from *Ruegeria pomeroyi* in a dynamic kinetic resolution reaction of 2-phenylpropanal (Scheme 47).

**Scheme 48:**
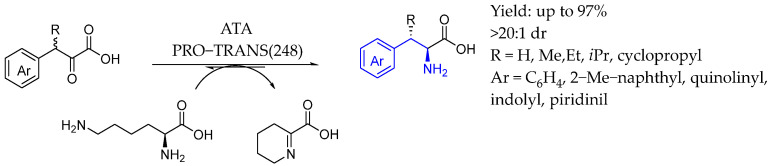
Chiral α-amino acid-containing AEA by Dunham et al. [97].

Also in 2024, Dunham et al. [97] utilized lysine as amine donor in transaminase-catalyzed dynamic kinetic resolution reactions to produce β-branched arylalanines. Their mechanistic study revealed that the ketone byproduct derived from lysine readily cyclized into a six-membered imine, effectively driving the equilibrium toward the desired product without requiring excessive amounts of the amine donor or a multi-enzyme cascade. This method successfully yielded a wide range of β-branched arylalanines achieving high yields and excellent selectivity (Scheme 48).

**Scheme 49:**
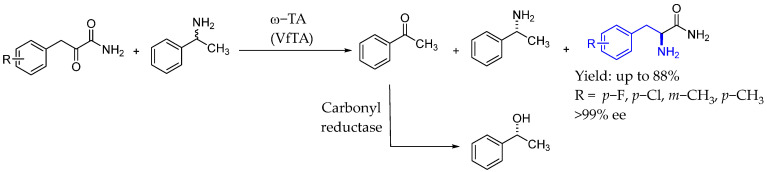
One-pot cascade reaction to produce chiral AEA enabled by transaminase catalysis by Wang et al. in 2024 [98].

Finally, Wang et al. [98] established a revolutionary and sophisticated one-pot cascade reaction using a two-enzyme catalytic protocol which allowed for the incorporation of racemic amines as amino donors, leading to the production of products with remarkable levels of ee > 99%. By producing chiral alcohols from ketone byproducts and converting racemic amines into chiral amines through kinetic resolution, this approach was able to push the enzymatic reaction equilibrium to the product formation side, which efficiently addressed the costs associated with the production of desired chiral amines (Scheme 49).

### 5.2. Amino Dehydrogenase

**Scheme 50:**
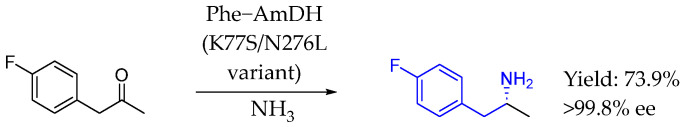
Amine dehydrogenase-based synthesis of chiral AEA by Bommarius et al. [99].

In 2013, Bommarius et al. [99] described the development of a highly active amine dehydrogenase derived from a phenylalanine dehydrogenase scaffold, which lacked original amine dehydrogenase activity. That engineered enzyme could accept both aliphatic and benzylic ketone substrates and demonstrated exceptional stereoselectivity (>99.8% ee), retaining the high selectivity of its wild-type counterpart. Thus, chiral AEA could be synthesized from the corresponding ketone-based substrates (Scheme 50).

**Scheme 51:**
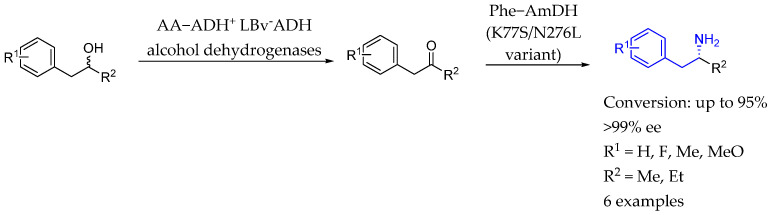
Chiral AEA synthesis by alcohol conversion into amine products by Turner et al. in 2015 [100].

In 2015, Turner et al. [100] described a biocatalytic method for converting alcohols into enantiopure amines using a dual-enzyme hydrogen-borrowing cascade. That approach utilized an alcohol dehydrogenase in tandem with an amine dehydrogenase to efficiently aminate a diverse range of primary and secondary alcohols, achieving a conversion of up to 96% conversion and a 99% enantiomeric excess, when synthesizing chiral AEA. The process was redox self-sufficient, featuring high atom efficiency by sourcing nitrogen from ammonium and producing only water as a byproduct, making it an environmentally friendly option for industrial applications (Scheme 51).

**Scheme 52:**
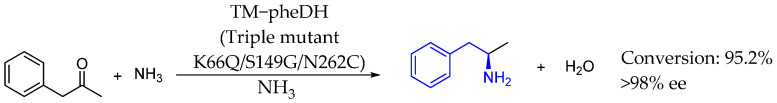
New amino dehydrogenase used by Li et al. in 2015 to produce chiral AEA [101].

Also in 2015, Li et al. [101] designed a new amine dehydrogenase, termed TM_pheDH, derived from Rhodococcus phenylalanine dehydrogenase (pheDH) to enable the asymmetric reductive amination of phenylacetone, producing (*R*)-amphetamine with an over 98% enantiomeric excess. That enzyme was a significant addition to the limited family of amine dehydrogenases, enhancing the asymmetric reductive amination of ketones, crucial for sustainable pharmaceutical production (Scheme 52).

**Scheme 53:**
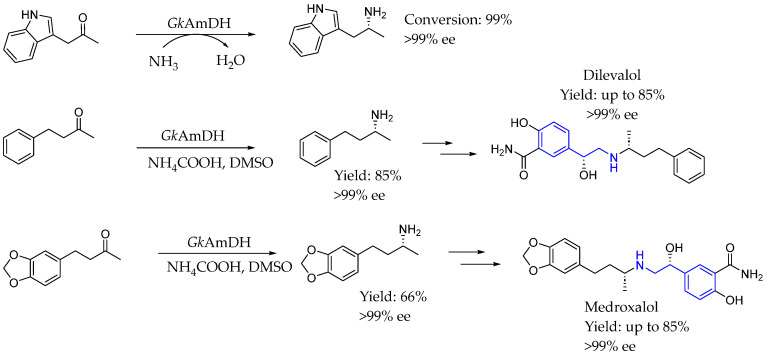
Amino dehydrogenase new scope to synthesize dilevalol and medroxalol by Chen et al. [102].

Years later, in 2021, Chen et al. [102] expanded the substrate range of the engineered GkAmDH from *Geobacillus kaustophilus* through laboratory evolution, enabling the synthesis of diverse bulky chiral amines with a conversion and ee greater than 99%. Notably, they produced two key chiral intermediates for the drugs medroxalol and dilevalol at a gram scale, achieving yields of up to 85% and ee > 99% (Scheme 53).

**Scheme 54:**
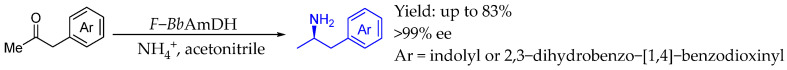
Shield machine-like substrate used by Mu et al. to produce chiral AEA [103].

Later on, in 2024, Mu et al. [103] reported a shield machine-like substrate walking strategy to quickly evolve F-BbAmDH from *Bacillus badius* for accessing the difficult-to-aminate fused-ring and linked-ring aryl ketones. Thus, chiral AEAs were synthesized from the corresponding ketone derivatives (Scheme 54).

**Scheme 55:**
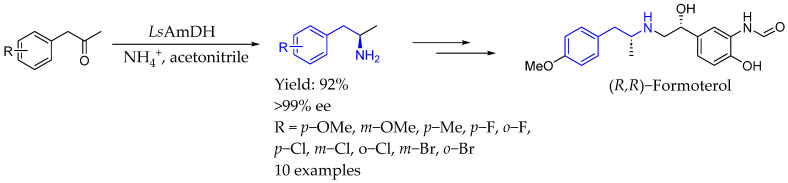
Reductive amination of ketones enabled by amino dehydrogenase to produce chiral AEA by Zhy et al. [104].

Also in 2024, Zhu et al. [104] reported the biocatalytic reductive amination of ketones using an engineered amine dehydrogenase (AmDH) mutant W5–5, which allowed for the synthesis of chiral primary amines, specifically amphetamine analogs, at low ammonium concentrations. That approach enhanced yields and enantioselectivity (ee values > 99%) while minimizing ammonium waste, contributing to a more sustainable and economical process (Scheme 55).

### 5.3. Imine Reductases

**Scheme 56:**
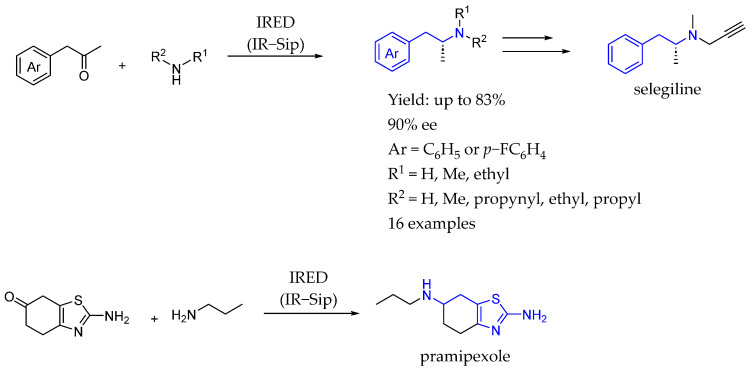
IRED used by Hörne et al. in 2017 [105] to produce chiral AEA from the corresponding ketone derivatives.

**Scheme 57:**
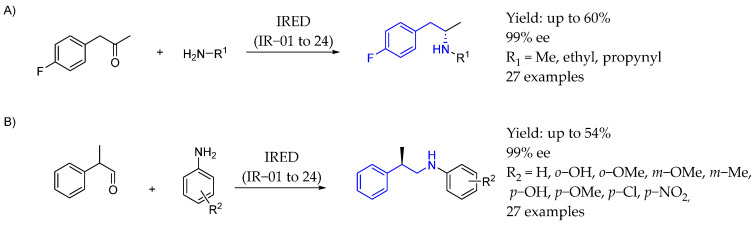
Chiral synthesis by Roiban et al. [106] enabled by IRED catalysis in 2017 (**A**) and 2019 (**B**).

In 2017, Höhne et al. presented [105] the one-step asymmetric synthesis of (*R*)- and (*S*)-rasagiline via reductive amination using imine reductases (Scheme 56). The process was based on the use of iminoreductase catalysis in order to produce primary amines from the corresponding ketone derivatives. That way, chiral AEA was synthesized with a good yield and moderate ee. The same methodology was also used in the same year by Roiban et al. [106] to synthesize the same product while expanding the imine reductase toolbox, adding a couple of amino substituents (Scheme 57A). Following their studies in iminoreductase-based biocatalysis synthesis, in 2019 [107], they developed the synthesis of β-chiral amines by the dynamic kinetic resolution of α-branched aldehydes applying the imine reductase from Streptomyces, facilitating the corresponding *N*-methylated β-chiral amines, which were obtained with a >95% conversion and high ee (Scheme 57B).

**Scheme 58:**
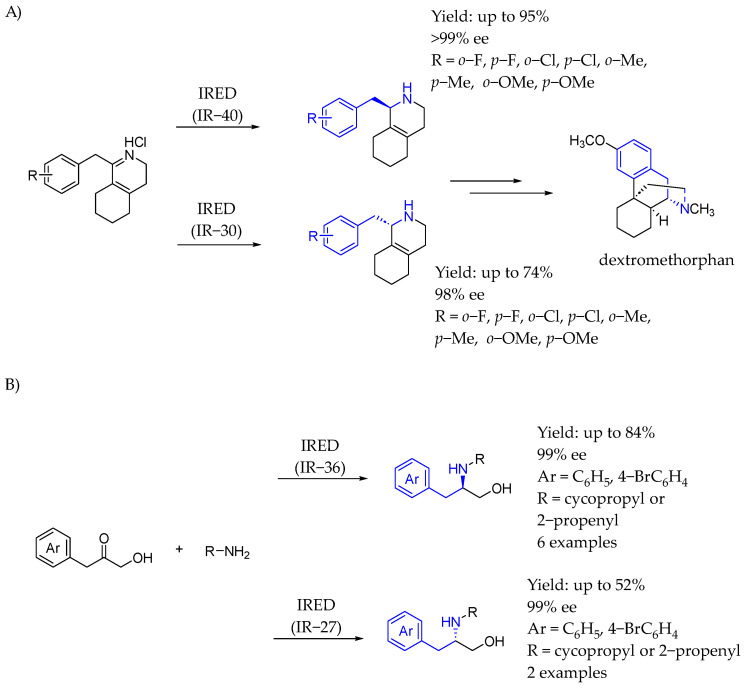
Chiral synthesis of dextromethorphan intermediates (**A**) and chiral amino alcohol-containing AEA synthesis (**B)** enabled by IRED by Zhu et al. in 2019 and 2022, [108,109] respectively.

Also in 2019, Zhu et al. [108] provided an effective route to synthesize a pharmaceutically important morphinan skeleton via the enantioselective reduction of bulky α,β-unsaturated imine precursors by imine reductase-catalyzed enantioselective reduction, from *Sandarearacinus amylolyticus* (IR40) (Scheme 58A). The key intermediate which could be synthesized through this methodology was an interesting chiral AEA which could be derivatized in order to obtain the desired bioactive compound. Subsequently, in 2022 [109], they developed a one-pot, two-step enzymatic process involving the hydroxymethylation of aldehydes catalyzed by benzaldehyde lyase, followed by asymmetric imine reductase catalysis (Scheme 58B). That method provided an environmentally friendly and economical approach to produce chiral *N*-substituted 1,2-amino alcohol-based AEA from readily available simple aldehydes and amines. The synthesis achieved excellent enantiomeric excess values ranging from 91% to 99%, with moderate to high yields of 41% to 84%.

**Scheme 59:**
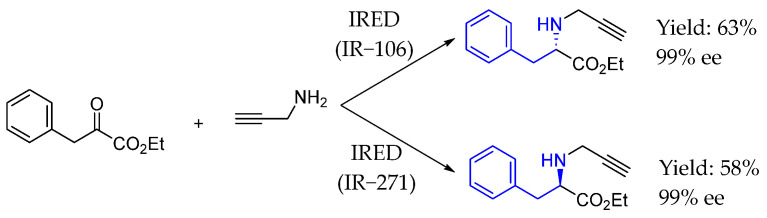
IRED used for the synthesis of chiral AEA from α-ketoesters by Turner et al. in 2021 [110].

Then, in 2021, Turner et al. [110] developed a highly efficient biocatalytic strategy for the synthesis of *N*-substituted amino esters from α-ketoesters and amines catalyzed by IREDs. That approach offered efficient access to various enantiomerically pure *N*-substituted amino esters from aryl- and alkyl-substituted α-ketoesters with exquisite and complementary enantioselectivities, addressing the problems identified in previous chemocatalytic and biocatalytic approaches to attain these compounds (Scheme 59).

**Scheme 60:**
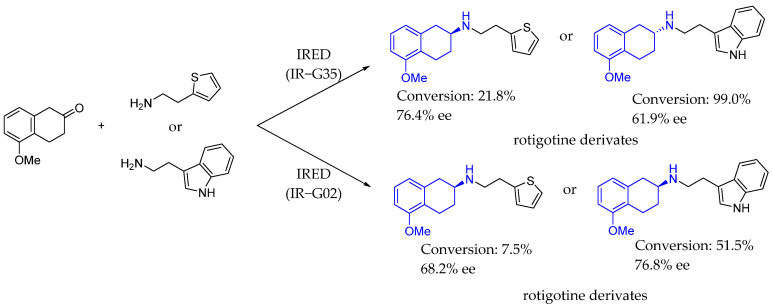
Rotigotine and derivatives synthesized by Wang et al. in 2022, [111] enabled by IRED catalysis.

In 2022, Wang et al. [111] reported the potential of specific IREDs (IR-G02 and 35) for expanding the biocatalyst toolbox for chiral amine synthesis. Using increasing molecule-volume screening, these IREDs were identified as capable of accepting bulky amine substrates. Notably, IR-G02 demonstrated an excellent substrate scope, enabling the synthesis of various amines and active pharmaceutical ingredient (API) substructures. IR-G35, for instance, achieved moderate enantioselectivity (>77%) in synthesizing a rotigotine substructure. Overall, these IREDs offer a promising biological pathway for producing medicinal chiral amines (Scheme 60).

**Scheme 61:**
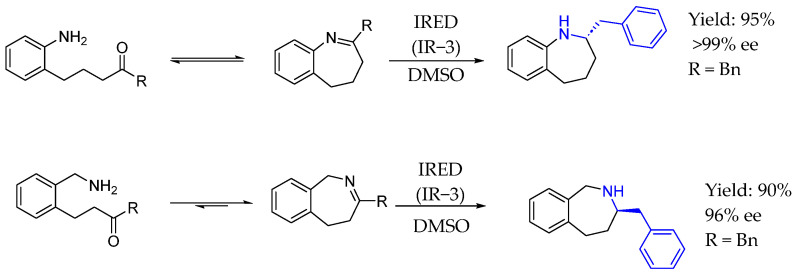
Benzazepine-containing AEA by IRED catalysis synthesized in 2023 by Husain et al. [112].

Later on, in 2023, Husain et al. [112] showed for the first time that imine reductases could be used for the asymmetric synthesis of tetrahydro-1-, 2-, and 3-benzazepines via the direct reduction of seven-membered cyclic imines to access a broad range of variously substituted tetrahydro benzazepines in high yield and high enantiomeric access. In this process, an interesting chiral AEA was synthesized, in which the nitrogen atom was embedded into a benzazepine ring (Scheme 61).

**Scheme 62:**
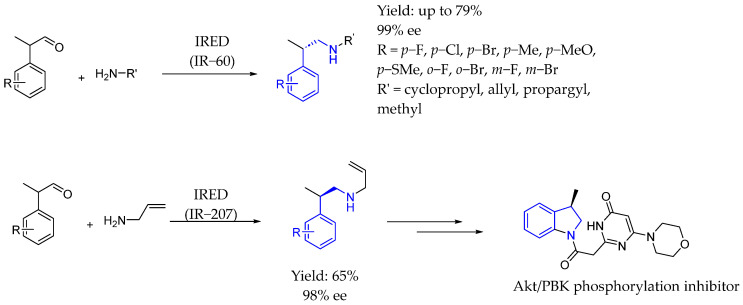
IRED-catalyzed synthesis of β-aryl propanamines by Yao et al. [113].

In 2023, Yao et al. [113] reported a stereocomplementary synthesis of β-aryl propanamines through enzymatic dynamic kinetic resolution and reductive amination, achieving excellent enantiomeric excess values and high yields when it came to the synthesis of chiral AEA. The IREDs showed high stereoselectivity in the reductive amination of 2-phenylpropanal with different amines. Also, they applied the synthesis of the aforementioned products by derivatizing them, producing a phosphorylation inhibitor through intramolecular Buchwald–Hartwig cross-coupling and deallylation, providing an effective method to construct this class of pharmaceutically important compounds (Scheme 62).

### 5.4. Photobiocatalysis

**Scheme 63:**
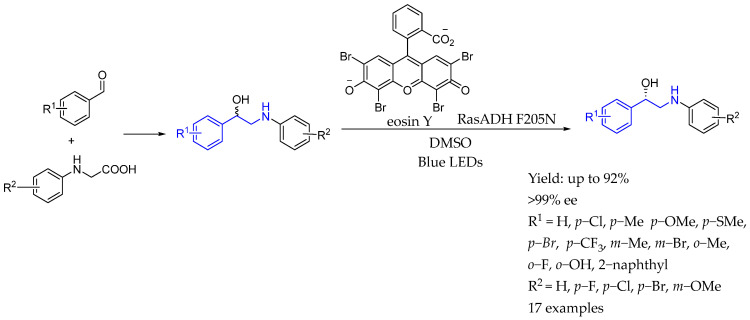
Chiral amino alcohol-containing AEA by Zhu et al. in 2023 [114].

In 2023, Zhu et al. [114] designed a redox-neutral photocatalysis and enzymatic catalysis system for the asymmetric synthesis of chiral 1,2-amino alcohols through the decarboxylative radical C–C coupling of *N*-arylglycines and aldehydes. The approach combined the organic photocatalyst eosin Y with carbonyl reductase RasADH, facilitating efficient enantioenriched product formation. The proposed reaction mechanism highlighted the dual role of eosin Y, which aided in both the synthesis of racemic amino alcohols and the deracemization process, while the carbonyl reductase ensured control over enantioselectivity (Scheme 63).

**Scheme 64:**
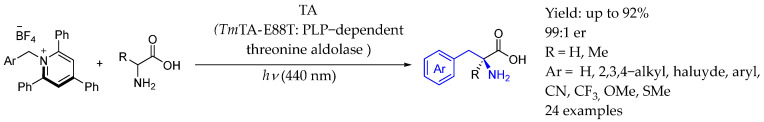
Photobiocatalyzed synthesis of chiral AEA by Yang et al. in 2024 [115,116].

In 2024, Yang et al. [115,116] reported a photobiocatalytic asymmetric sp^3^–sp^3^ oxidative cross-coupling reaction which utilized engineered pyridoxal biocatalysts, photoredox catalysts, and an oxidizing agent. This process produced a variety of α-tri- and tetrasubstituted non-canonical amino acids with up to two contiguous stereocenters in a controlled enantio- and diastereoselective manner. Furthermore, they developed a visible light-driven, threonine aldolase-catalyzed enantioselective α-alkylation of amino acids using Katritzky salts as the radical precursors. Surprisingly, this last photobiocatalytic process did not require the use of photoredox catalysts and operated through an unconventional photoinduced radical generation involving a PLP-derived aldimine (Scheme 64).

**Scheme 65:**
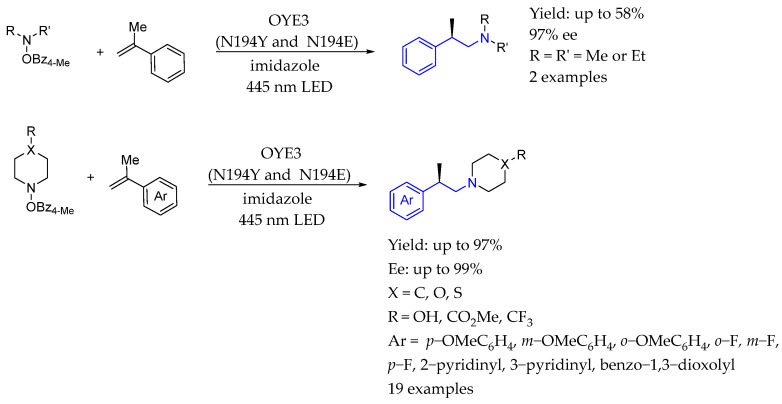
Chiral AEA synthesis by Zhao et al. in 2024 [117] enabled by photobiocatalysis.

Also in 2024, Zhao et al. [117] presented an application of photoenzymatic catalysis for asymmetric intermolecular hydroamination, utilizing an engineered flavin-dependent ene-reductase. The enzyme photocatalytically generated the iminium radical cation from hydroxylamines, facilitating the asymmetric hydroamination to produce enantioenriched tertiary amines via enzyme-mediated hydrogen atom transfer (Scheme 65).

### 5.5. Others

#### 5.5.1. Reductive Aminases

**Scheme 66:**
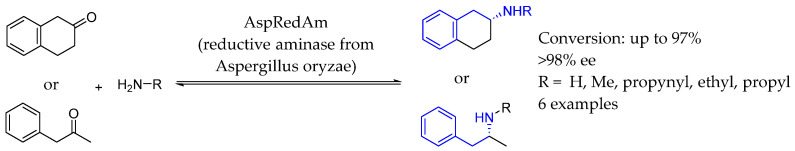
Reductive aminase used for the synthesis of chiral AEA by Turner et al. in 2017 [118].

In 2017, Turner et al. [118] discovered an NADP(H)-dependent reductive aminase from *Aspergillus oryzae* (AspRedAm) that efficiently catalyzed the coupling of various carbonyl compounds with primary and secondary amines, achieving high conversion rates and stereoselectivity (up to 98%). Kinetic studies confirmed AspRedAm’s ability to catalyze both imine formation and reduction, positioning it as a promising biocatalyst for synthesizing industrially relevant AEAs (Scheme 66).

#### 5.5.2. Lyases

**Scheme 67:**
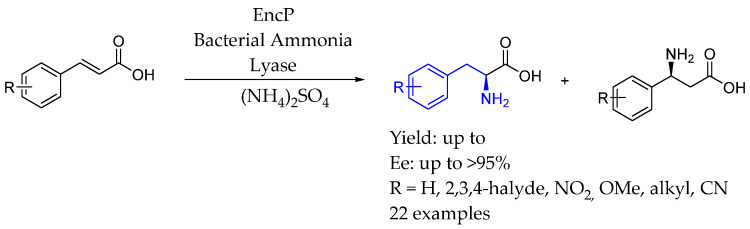
Chiral synthesis of AEA enabled by ammonia lyase in 2015, by Turner et al. [119].

In 2015, Turner et al. [119] investigated the ammonia lyase EncP from *Streptomyces maritimus* as a biocatalyst for producing (*S*)-β-arylalanines, complementing existing methods using phenylalanine ammonia lyases (PALs) and phenylalanine aminomutases (PAMs). EncP converted arylacrylates into (*S*)-α- and (*S*)-β-arylalanines, with regioselectivity influenced by the electronic properties of the substrates. The results showed that EncP could be effectively optimized for enhanced regioselectivity in ammonia addition to cinnamic acid derivatives, achieving high selectivity for either (*S*)-β or (*S*)-α arylalanines (Scheme 67).

**Scheme 68:**
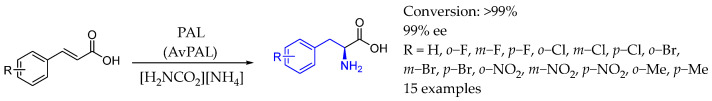
Phenyl alanine lyase in combination with amino acid decarboxylase to produce chiral AEA by Flitch et al. in 2019 [120].

In 2019, Flitsch et al. [120] developed and optimized a two-enzyme system for synthesizing β-phenylethylamine derivatives from readily available ring-substituted cinnamic acids. The method used a combination of phenyl alanine lyase PAL and amino acid decarboxylase AADC enzymes, both specific to the amino acid intermediate. This biocatalytic system allowed a streamlined, one-pot transformation without the need for chromatographic purification or complex separation steps (Scheme 68).

#### 5.5.3. Aminotransferases

**Scheme 69:**
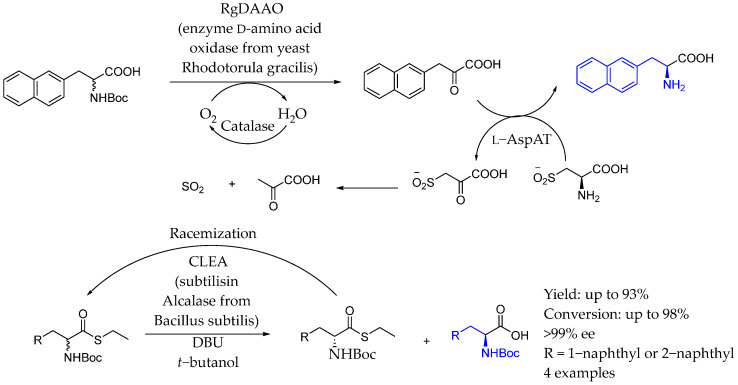
Aminotransferase-catalysis used for the synthesis of chiral AEA by Servi et al. in 2012 [121].

In 2012, Servi et al. [121] developed a double catalyst system (protease + base) for the dynamic kinetic resolution of isomeric 1- and 2-α-naphthyl glycines and -alanines by leveraging the in situ racemization of their thioesters. They optimized experimental conditions based on the different C-acidity of the compounds to achieve simultaneous resolution and racemization, showing excellent yields and nearly complete stereoselectivity for racemic N-protected naphthyl amino acid-thioesters. The double catalysis was enabled by D-amino acid oxidase, producing the corresponding keto-acid, and subsequent chiral amination by L-specific-aspartate amino transferase, achieving asymmetric AEA (Scheme 69).

#### 5.5.4. Aldoxime Dehydratase

**Scheme 70:**
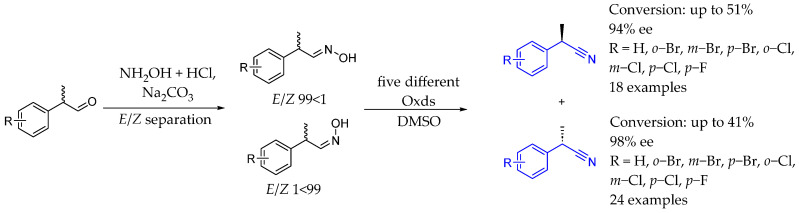
Aldoxime dehydratase-catalysis in order to produce a wide range of chiral AEAs by Groger et al. in 2017 [122].

In 2017, Groger et al. [122] developed a cyanide-free technology for the synthesis of chiral nitrile-containing AEA through the biocatalytic enantioselective dehydration of a wide range of aldoximes. Using efficient recombinant *E. coli* whole-cell catalysts that expressed aldoxime dehydratases, the method achieved a high enantiomeric excess (over 90%, up to 99% in some cases). A noteworthy finding of the research was the observed enantiospecificity: depending on whether the *E* or *Z* isomer of the racemic aldoxime substrate was employed, the same enzyme preferentially produced one of the available enantiomers of the corresponding nitrile (Scheme 70).

#### 5.5.5. Cytochrome c

**Scheme 71:**
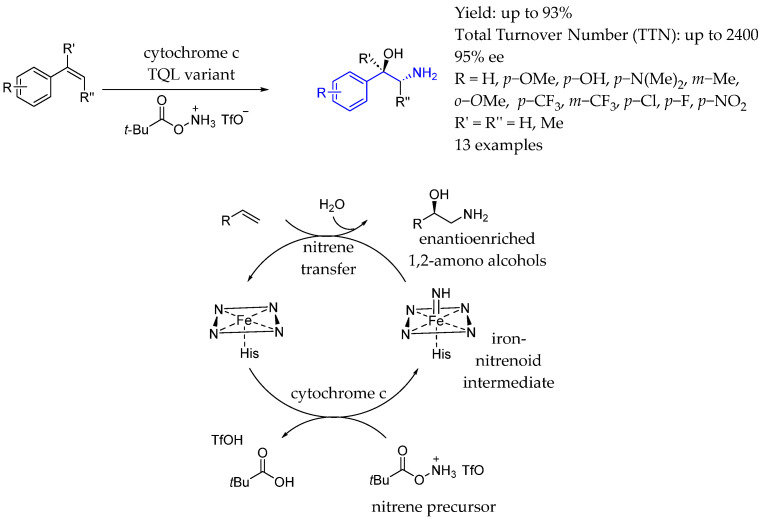
Methodology used to produce polysubstituted amino alcohol-containing chiral AEA enabled by cytochrome c by Arnold et al. in 2019 [123].

Through directed evolution, in 2019, Arnold et al. [123] engineered this thermostable cytochrome c biocatalyst to transform alkenes into amino alcohols with high enantioselectivity (90% ee) under anaerobic conditions, using *O*-pivaloyl hydroxylamine as the aminating agent. The reaction was believed to proceed via a reactive iron–nitrogen species formed in the enzyme’s active site, allowing for adjustments in the catalyst’s activity and selectivity through protein engineering (Scheme 71).

#### 5.5.6. Amino Acid Decarboxylase

**Scheme 72:**
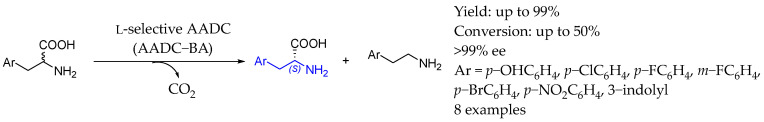
One-pot D-amino acid synthesis by Shin et al. [124] using amino acid decarboxylase.

In 2022, Shin et al. [124] reported a method for one-pot production of enantiopure D-amino acids and neuroactive monoamines through the kinetic resolution of aromatic amino acids using aromatic L-amino acid decarboxylase from *Bacillus atrophaeus* (AADC-BA). That enzyme, with broad substrate specificity and high enantioselectivity, achieved enantiomeric excess of D-amino acids of over 99% at a conversion of racemic substrates of around 50%. To enhance efficiency, an in situ product removal strategy using cation exchange resin was implemented to remove inhibitory monoamines selectively (Scheme 72).

#### 5.5.7. Ketoreductase

**Scheme 73:**
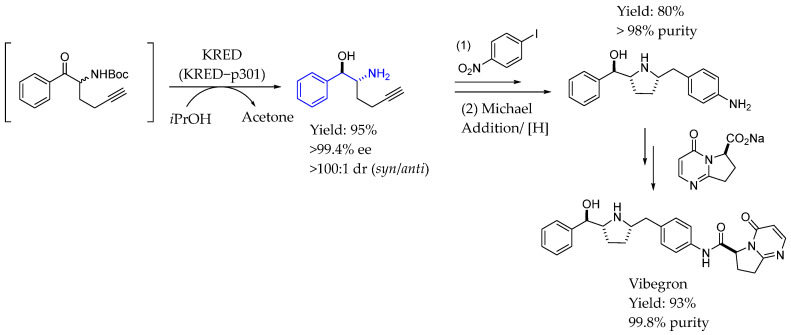
Enzymatic DK reduction by ketoreductase to produce chiral AEA by Xu et al. [125].

In 2018, Xu et al. [125] described a highly efficient, asymmetric synthesis of vibegron by an enzymatic DK reduction to establish the stereochemistry in excellent enantio- and diastereoselectivity. Incorporating structurally designed mutations into directed enzyme evolution produced a suitable ketoreductase and the subsequent intramolecular Michael addition followed by diastereoselective hydrogenation concisely constructed the backbone of vibegron (Scheme 73).

#### 5.5.8. Norcoclaurine Synthase

**Scheme 74:**
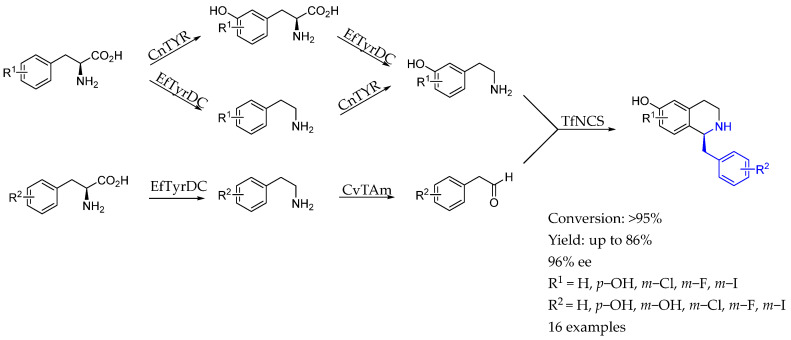
Styrosinase, tyrosine decarboxylase, transaminase, and norcoclaurine synthase to produce chiral AEA, by Hailes et al. [126].

In 2022, Hailes et al. [126] combined styrosinase, tyrosine decarboxylase (EfTyrDC), transaminase (CvTAm), and norcoclaurine synthase (TfNCS) in a parallel enzyme cascade to produce halogenated benzylisoquinoline alkaloids with a high enantiomeric excess. The approach allowed for unique combinations of arylethylamines and aldehydes to synthesize double-halogenated BIAs for the first time, showcasing the versatility of enzyme cascades in alkaloid synthesis (Scheme 74).

## 6. Conclusions

The 2-arylethylamine motif is present in multiple natural and design bioactive compounds due to its interesting properties when it comes to interacting with the Central Neural System. The chiral synthesis of AEA has been proved to be a challenge for the scientific community. We presented the last advances in the synthesis of the aforementioned compounds, considering the methodology to obtain the chiral moiety towards the production of asymmetric AEA in C-1, C-2, or in both carbon atoms. Chiral induction catalysis, organocatalysis, organophotocatalysis, and enzymatic catalysis were discussed, pointing to the most relevant advances and procedures and considering the yield, ee, dr, and/or scope availability, when possible. Regarding chiral induction, a short but concise list of studies was chronologically listed. As for organocatalysis, different chiral auxiliaries were taken into consideration and studies were discussed considering the corresponding organocatalyst, namely, thioureas, phosphoric acid and phosphonates, amines, amides and ammonium species, aryl iodine species, aldehydes, boron species, and selenium species. When it comes to organophotocatalysis, precise methodologies using light-sensitive organocatalysts were discussed. Finally, studies involving enzymatic catalysis were listed based on the corresponding enzyme enabling the desired product, transaminases, amino dehydrogenase, and iminoreductase catalysis being the most successful methodologies presented in this work. Also, DFT studies, DKR, and interesting derivatization towards bioactive compounds were presented, when relevant.

Many of the methods described allow the obtainment of both primary and secondary amines, whose manipulation and greater functionalization herald great potential for the development of derivatives for application in the pharmaceutical industry. The large quantity of data provided and active compounds accessible, with the help of artificial intelligence (AI), highlight the possibility of significant progress in the design of new catalysts, optimization of reaction conditions, and prediction of biological activity with important benefits in relation to the acceleration of discovery, greater selectivity, cost reduction, and greater molecular diversity. Despite the great potential of AI-assisted asymmetric AEA synthesis, future challenges are the need for large quantities of data, the interpretability of AI models, and the experimental validation of predictions. Additionally, the biological applications of chiral AEAs, especially in pharmaceuticals targeting neurological disorders, present promising avenues for further investigation, combining computational and experimental approaches to streamline drug development. We hope that this review can help the scientific community thanks to the extensive methodological development presented regarding the synthesis of interesting moieties when it comes to the obtention of bioactive compounds with important pharmacological activity.
